# ELF-MF exposure affects the robustness of epigenetic programming during granulopoiesis

**DOI:** 10.1038/srep43345

**Published:** 2017-03-07

**Authors:** Melissa Manser, Mohamad R. Abdul Sater, Christoph D. Schmid, Faiza Noreen, Manuel Murbach, Niels Kuster, David Schuermann, Primo Schär

**Affiliations:** 1 Department of Biomedicine, University of Basel, Mattenstrasse 28, Basel, CH-4058, Switzerland; 2 Swiss Tropical and Public Health Institute, Socinstrasse 57, Basel, CH-4002, Switzerland; 3 University of Basel, Petersplatz 1, Basel, CH-4001, Switzerland; 4 SIB Swiss Institute of Bioinformatics, Basel, Switzerland; 5 IT’IS Foundation, Zeughausstrasse 43, Zürich, CH-8004, Switzerland; 6 Swiss Federal Institute of Technology (ETH), Zürich, CH-8006, Switzerland

## Abstract

Extremely-low-frequency magnetic fields (ELF-MF) have been classified as “possibly carcinogenic” to humans on the grounds of an epidemiological association of ELF-MF exposure with an increased risk of childhood leukaemia. Yet, underlying mechanisms have remained obscure. Genome instability seems an unlikely reason as the energy transmitted by ELF-MF is too low to damage DNA and induce cancer-promoting mutations. ELF-MF, however, may perturb the epigenetic code of genomes, which is well-known to be sensitive to environmental conditions and generally deranged in cancers, including leukaemia. We examined the potential of ELF-MF to influence key epigenetic modifications in leukaemic Jurkat cells and in human CD34+ haematopoietic stem cells undergoing *in vitro* differentiation into the neutrophilic lineage. During granulopoiesis, sensitive genome-wide profiling of multiple replicate experiments did not reveal any statistically significant, ELF-MF-dependent alterations in the patterns of active (H3K4me2) and repressive (H3K27me3) histone marks nor in DNA methylation. However, ELF-MF exposure showed consistent effects on the reproducibility of these histone and DNA modification profiles (replicate variability), which appear to be of a stochastic nature but show preferences for the genomic context. The data indicate that ELF-MF exposure stabilizes active chromatin, particularly during the transition from a repressive to an active state during cell differentiation.

The increasing use of electronic appliances generating electromagnetic fields in the extremely-low-frequency range of 50 or 60 Hz (ELF-MF) has raised concerns about potential health risks. The main sources of ELF-MFs are in-house installations, household appliances and powerlines, resulting in average indoors exposure levels between 0.025 and 0.07 μT in Europe^1^,^2^. Based on epidemiological studies that associated ELF-MF exposure with an increased risk for childhood leukaemia, ELF-MFs were categorized as being possibly carcinogenic to humans (group 2B) by the International Agency for Research on Cancer (IARC)^3^,^4^. Animal and cellular studies, performed to address biological effects of ELF-MF exposure and to pinpoint mechanisms underlying potential health impacts, however, failed to provide a consistent mechanistic explanation of these epidemiological observations^1^,^3^. Most animal studies did not support evidence that magnetic fields can cause tumours, exceptions being recent reports indicating a co-carcinogenic effect in rats exposed to sinusoidal 50 Hz ELF-MF in combination with acute low-dose γ-ray exposure^5^ or formaldehyde^6^.

Acute lymphoblastic leukaemia (ALL) is the most common type of childhood leukaemia, characterized by accumulation of T or B lymphocytes in progenitor stages, unable to terminally differentiate^7^,^8^. Many ALLs arise from foetal genetic lesions or translocations like TEL-AML1 (ETV6-RUNX1) or MLL-TET1 fusions in blood progenitor cells, resulting in unlimited self-renewal and failure in stage-specific developmental arrest^9^. Besides the foetal genetic events, the classical ‘two-hit’ model of childhood ALL postulates a requirement of a second hit after birth in the form of additional chromosomal or genetic alteration^10^. Prominent amongst these appear to be mutations in genes encoding epigenetic modifiers like in the methyltransferase EZH2, a subunit of the polycomb repressive complex 2, or the DNA methyltransferase DNMT3a^11^,^12^,^13^. These mutations in epigenetic modifiers indicate that defects in the control of cell differentiation-associated changes in gene expression and chromatin landscapes contribute to the establishment of ALL.

Cancers generally emerge as a consequence of progressive change in genome structure and function, including mutation of the DNA sequence and alteration of chromatin structure and gene expression^14^. Genomic instability is therefore a hallmark of tumour progression^15^,^16^. Whether or not ELF-MFs have the power to induce genetic mutations is questionable as the energy deposited by ELF-MFs is orders of magnitudes lower than would be required to affect chemical bonds in DNA^17^. Therefore, notwithstanding occasional reports of a genotoxic potential of ELF-MFs^18^,^19^,^20^,^21^, it seems unlikely that ELF-MF-induced genetic mutations contribute significantly to the mutagenesis in cancer. Another hallmark of cancers are aberrations in the cell type-specific patterns of epigenetic modifications. Epigenetic modifications to histone proteins and the DNA, established mainly during cell differentiation, guide and stabilize cell-type-specific gene expression. This “programming” of genomes in differentiating cells is instructed by environmental cues and, hence, is also likely to be sensitive to disturbance by environmental factors^22^,^23^, such as EMFs. Consistent with a possible impact of ELF-MF exposure on epigenetic cell programming, it has been reported that ELF-MFs are able to alter neural differentiation^24^,^25^,^26^,^27^. Regulatory epigenetic modifications include the acetylation and methylation of histone tails and the methylation of DNA cytosine bases^28^,^29^, altogether establishing three main classes of chromatin; i.e. active, repressed and poised chromatin. Active chromatin, comprising highly expressed genes, is marked by trimethylation of histone 3 lysine 4 (H3K4me3), acetylation of histone 3 lysine 27 (H3K27ac) and unmethylated DNA cytosine, whereas chromatin correlating with gene repression is characterized by histone 3 trimethylation at lysine 27 (H3K27me3) and lysine 9 (H3K9me3) and DNA cytosine methylation (5-methylcytosine, 5mC). Transcriptionally poised chromatin is co-occupied by the active and repressive marks H3K4me2/3 and H3K27me3 and is located preferentially at developmental genes in stem cells^30^. The potential of ELF-MF exposure to destabilize epigenetic modifications in general and in a cancer-relevant manner has not been addressed systematically. Yet, it was reported to alter global levels of 5mC and the expression of DNA methyltransferases DNMT1 and DNMT3b in murine spermatocyte-derived cells^31^, and to increase the reprogramming efficiency of somatic cells by upregulation of the histone lysine methyltransferase Mll2, which appears to enrich H3K4me3 at pluripotency genes^32^.

To investigate whether ELF-MFs have the potential to alter the epigenome, we analyzed the impact of exposure on the stability of key active (H3K4me2) and repressive (H3K27me3) histone modifications in a leukaemic cell line, as well as on the formation of cell-type-specific H3K4me2 and H3K27me3 and DNA methylation patterns during *in vitro* differentiation of human cord blood cells under highly controlled and standardized conditions. We profiled these modifications genome-wide and found that ELF-MF exposure has no reproducible effects on the pattern of histone and DNA modifications in leukaemic cells nor on the establishment of cell-type-specific modifications during haematopoietic differentiation. Yet, the ELF-MF exposure appeared to affect the variability, i.e., the robustness and reproducibility of the epigenetic marks in replicate cell populations.

## Results

### ELF-MF exposure of Jurkat cells does not induce reproducible alterations in histone modifications

**Figure 1:**
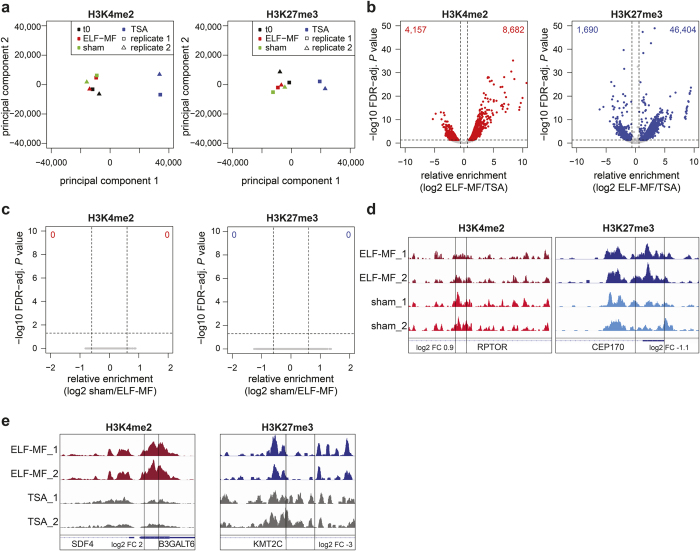
ELF-MF exposure does not alter global patterns of histone modifications in Jurkat cells. Jurkat cells were ELF-MF- (50 Hz sinus, 1 mT, 5′ on/10′ off), sham-exposed or treated with 10 nM Trichostatin A (TSA) for 72 h. Profiles of H3K4me2 and H3K27me3 modification were generated by ChIP-sequencing of two replicate (representing pools of three biological replicates). (**a**) Principal component analysis of H3K4me2 and H3K27me3 ChIP-seq data from non-exposed (t0), ELF-MF- and sham-exposed or TSA-treated cells, comparing read counts in 500 bp genomic tiles. (**b**,**c**) Comparison of H3K4me2 and H3K27me3 read counts within 500 bp genomic tiles between TSA-treated and ELF-MF-exposed (**b**) or ELF-MF- and sham-exposed (**c**) cells. Shown are differences in relative enrichments as log2-fold change (FC) (x-axis) against the false discovery rate (FDR)-adjusted *P* value (likelihood ratio test) (y-axis). Statistically significant tiles (FC > ±0.6, FDR-adjusted *P* < 0.05) are highlighted in red (H3K4me2) or blue (H3K27me3). (**d**) Exemplary profiles of H3K4me2 and H3K27me3 modifications at the *RPTOR* and *CEP170* locus with FCs between sham- and ELF-MF-exposed cells of 1.8 and 2.1, respectively, but not reaching statistical significance. (**e**) H3K4me2 and H3K27me3 profiles at the *B3GALT6* and *KMT2C* locus, significantly different in ELF-MF- and TSA-treated cells.

First, we determined if ELF-MF exposure affects the epigenetic stability of the T cell lymphoma cell line Jurkat, i.e., induces consistent alterations of epigenetic modification patterns in replicate exposure experiments. We profiled the key histone marks H3K4me2 and H3K27me3 (Fig. 1) in Jurkat cells, exposed intermittently (5′ on/10′ off) to either a 50 Hz sine ELF-MF at a flux density of 1 mT, or to a sham control (<7 μT residual field) for 72 h (Supplementary Fig. S1). Exposure settings were blinded throughout the experiments. We also included a treatment with the histone deacetylation inhibitor trichostatin A (TSA) at a sub-toxic dose of 10 nM to assess the impact of a known epigenetic modulator (Supplementary Fig. S2). Previous studies – although not consistently – described effects of ELF-MF exposure on cell proliferation and apoptosis^18^,^33^,^34^, parameters that may influence epigenetic modifications on their own. So, we assessed exposure effects on proliferation, cell cycle progression and apoptosis in our exponentially growing Jurkat cell cultures. These controls revealed no significant exposure effect on either of these parameters throughout the experiment (72 h, approximately 3.5 cell cycles) (Supplementary Fig. S2). By contrast, TSA treatment (10 nM) resulted in a transient increase of G1 and a decrease of S and G2 phase cells 24 hours after treatment start without, however, affecting overall cell proliferation or the proportion of apoptotic cells. We therefore conclude that the ELF-MF exposure condition applied in our experiment did not affect cell viability and proliferation.

We then combined chromatin immunoprecipitation (ChIP) with next generation sequencing (ChIP-seq) to quantitatively map the occurrence of H3K4me2 and H3K27me3 in chromatin isolated from Jurkat cells before exposure (t0), following ELF-MF/sham exposure for 72 h, or treated with TSA. Two ChIP-seq replicates were generated from six biological replicates for each condition by pooling three samples each. About 51 million reads per ChIP-seq sample were mapped to the hg19 human genome. H3K4me2 and H3K27me3 profiles in 500 bp genomic tiles showed a high reproducibility between the replicates of all conditions (Supplementary Figs S3 and S4). Principal component analysis clearly separated TSA-treated Jurkat cells from exposed cells. ELF-MF- and sham-exposed cells, however, clustered together, indicating that the exposure had no global effect on H3K4me2 and H3K27me3 patterns (Fig. 1a). Consistently, we observed a large number of 500 bp tiles showing significantly different enrichment in H3K4me2 and H3K27me3 modifications in TSA-treated cells (FDR-adjusted *P* value < 0.05, log2-fold change [FC] > ±0.6) (Fig. 1b,e), but no significant differences were apparent when comparing ELF-MF- and sham-exposed Jurkat cells (Fig. 1c; Supplementary Fig. S5). Several loci, however, showed up to three-fold differential H3K4me2 and H3K27me3 occupancy upon ELF-MF exposure, as illustrated for two regions within the *RPTOR* and *CEP170* genes (Fig. 1d). These differences, however, did not reach statistical significance due to high variability. These results indicate that the global patterns of H3K4me2 and H3K27me3 modifications in leukaemic cells is largely unaffected by ELF-MF exposure.

### ELF-MF exposure does not reproducibly affect global patterning of histone marks in granulopoiesis

**Figure 2:**
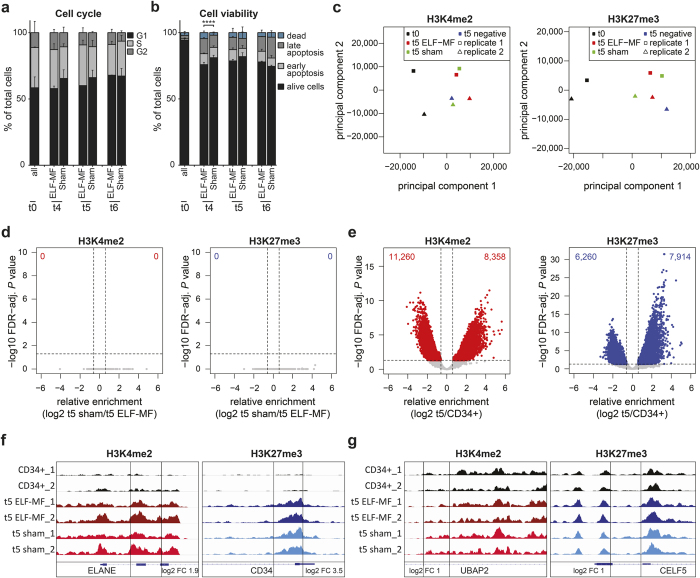
Patterning of histone modifications during granulopoiesis is not affected by ELF-MF. Human CD34+ cord blood cells were differentiated *in vitro* to neutrophilic progenitors under ELF-MF (50 Hz powerline signal, 1 mT, 5′ on/10′ off) or sham exposure for 5 days. (**a**,**b**) Cell cycle profiles and apoptosis were assessed by flow cytometry before and at days 4, 5 and 6 of neutrophilic differentiation and statistically analyzed by χ^2^ test on each replicate (****P < 0.001) and pairwise comparison by Student’s *t*-test. Shown are the mean percentage of cells in the G1, S, and G2 phase of the cell cycle (**a**) and of alive, early apoptotic, late apoptotic and dead/necrotic cells (**b**). Error bars; SEM of n ≥ 2 and n = 3 biological replicates of cell cycle and apoptosis analysis, respectively. (**c–g**) H3K4me2 and H3K27me3 profiles of CD34+ cells (t0) and neutrophilic progenitors (t5) were obtained by ChIP-seq and two replicates were statistically analyzed. (**c**) Principal component analysis of ChIP-seq data. (**d**,**e**) Comparison of ELF-MF- and sham-exposed neutrophilic progenitors (**d**) or of CD34+ and combined neutrophilic progenitor cells (**e**). Shown are differences in relative enrichments of ChIP-seq reads within 500 and 1,000 bp genomic tiles as log2-fold change (FC) (x-axis) against the false discovery rate (FDR)-adjusted *P* value (likelihood ratio test with variable dispersion) (y-axis). Statistically significant (FC > ±0.6, FDR-adjusted *P* < 0.05) tiles differentially occupied by H3K4me2 and H3K27me3 are highlighted in red and blue, respectively. (**f**,**g**) Exemplary profiles of H3K4me2 and H3K27me3 marks at genomic loci, identified by a 500 bp tile (black box) with significant differences in neutrophilic progenitors and CD34+ cells (**f**) or more than 2-fold enrichment between ELF-MF- and sham-exposed progenitor cells (**g**).

As epigenetic processes are highly dynamic during cellular differentiation^23^,^35^,^36^, we addressed whether ELF-MF exposure affects the differentiation-associated patterning of H3Kme2 and H3K27me3 marks. We differentiated *in vitro* CD34+ haematopoietic stem cells from human umbilical cord blood into the neutrophilic lineage by an established protocol^37^, either under exposure to a powerline-simulating ELF-MF (50 Hz, 1 mT, 5′ on/10′ off) or in parallel sham and no ELF-MF control conditions (Supplementary Fig. S1). The differentiation process was monitored by analysing granulocytic maturation stages by flow cytometry. Irrespective of the exposure condition, 90% of cells differentiated from the CD34+ progenitor state to a promyelocyte (75%) or myelocyte (15%) stage within five days (Supplementary Fig. S6). By day 10, more than 50% of cells matured into myelocytes and metamyelocytes/neutrophils, again without notable differences between ELF-MF- and sham-exposed populations. Also, cell proliferation was not affected by ELF-MF exposure (Supplementary Fig. S6). Throughout exponential growth, around 25% and 10% of cells in all populations were in S and G2 phase of the cell cycle, respectively (Fig. 2a; Supplementary Fig. S7). At days four and five (neutrophilic progenitor cell stage), ELF-MF-exposed cultures showed a small reduction of G1 and a compensating increase of S phase cells when compared to sham- and non-exposed controls. Investigating potentially associated effects of the ELF-MF exposure on cell viability as observed previously^33^,^34^,^38^,^39^, we quantified the proportion of alive cells, early apoptotic, late apoptotic and dead cells by flow cytometry. Expansion cultures of CD34+ cells (t0) were highly proliferating and composed of around 95% living cells (Fig. 2b; Supplementary Fig. S8). Following induction of differentiation, the fraction of apoptotic cells increased, establishing a significant difference between exposure conditions at day four, where 24% of cells were apoptotic or dead in ELF-MF-exposed cultures and 19% in sham-exposed cultures (Fig. 2b). Although small, these differences indicate that ELF-MF has the potential to induce apoptosis directly or indirectly in a small fraction of differentiating neutrophilic cells. The concomitant slight accumulation of ELF-MF-exposed cells in S phase suggest that this may be related to an ELF-MF-induced disturbance of S phase progression.

To determine the patterning of H3K4me2 and H3K27me3 modifications, we performed ChIP-seq with chromatin of cells harvested before (t0) and after five days (t5) of differentiation. Two ChIP-seq replicates were generated from three independent differentiation experiments by pooling experiments two and three into ChIP-seq replicate two. 26 million reads per ChIP-seq sample were mapped to the human genome, and read numbers in 500 and 1,000 bp genomic tiles were analyzed. The comparison of reads per tile between samples confirmed a good reproducibility and clearly separated samples of CD34+ cells and neutrophilic progenitors in a principal component analysis (Fig. 2c; Supplementary Figs S9 and S10). Comparing ELF-MF- with sham- and non-exposed cells after five days of differentiation, we first analyzed the ChIP-seq data by a likelihood-ratio test assuming fixed standard deviations. This identified only few genomic regions with differential (FDR-adjusted *P* value < 0.05, log2 FC > ±0.6) H3K4me2 or H3K27me3 enrichments (Supplementary Fig. S11), most pronounced in the comparison of ELF-MF-exposed with non-exposed cells. Nine and 19 genomic regions showed significant differences in H3K4me2 and H3K27me3 enrichment, respectively, between ELF-MF- and sham-exposed neutrophilic progenitors. To address potential false positives due the statistical reasons, we adjusted the analysis by allowing for variable standard deviations between samples. Analyzed this way, the differences in H3K4me2 or H3K27me3 occupancy between ELF-MF- or sham-exposed cells disappeared (FDR-adjusted *P* value < 0.05, log2 FC > ±0.6) (Fig. 2d). By contrast, comparing cells prior to and five days into differentiation yielded large numbers of tiles with significant differences in H3K4me2 and H3K27me3 occupancy (Fig. 2e), including loci like *ELANE* and *CD34* with 3.8- and 11-fold differential enrichments, respectively (Fig. 2f), documenting the epigenetic reorganization occurring throughout neutrophilic differentiation. Notably, despite the lack of significant differences between ELF-MF- and sham-exposed cells, many genomic loci showed substantial differential enrichments for either of the histone marks (up to 8- to 12-fold) (Fig. 2d), as illustrated in the respective profiles at the *UBAP2* and *CLF5* loci (Fig. 2g). These results show that, while the epigenetic landscape undergoes major alterations during neutrophilic differentiation, ELF-MF exposure does not reproducibly impair the global patterning of the two key histone modifications examined.

### Genome-wide formation of DNA methylation patterns is not affected by ELF-MF exposure

**Figure 3:**
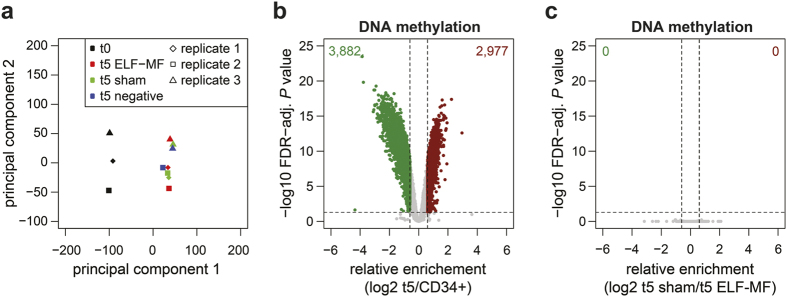
The DNA methylation pattern does not change upon ELF-MF exposure. DNA methylation of CD34+ human cord blood cells (t0) and neutrophilic progenitors (t5), *in vitro* differentiated under ELF-MF (50 Hz powerline signal, 1 mT, 5′ on/10′ off), sham or no exposure condition for five days, was analyzed by Illumina Infinium HumanMethylation 450 array. (**a**) Principal component analysis of all samples (M-values). (**b**,**c**) Differences in relative DNA methylation levels shown as log2-fold change (FC) (x-axis) are plotted against the false discovery rate (FDR)-adjusted *P* value (calculated by moderated *t*-statistics) (y-axis) for the comparison of (**b**) neutrophilic progenitors (combined progenitors of all exposure conditions) and CD34+ cells and (**c**) ELF-MF- and sham-exposed day 5 neutrophilic progenitors. Statistically significantly (FDR-adjusted *P* < 0.05) hypomethylated (FC < −0.6) and hypermethylated (FC > 0.6) CpGs are indicated in green and dark red, respectively.

Environmental conditions modulate epigenetic modifications not only at histones but also at the level of DNA^40^,^41^. To address whether ELF-MF affects DNA cytosine methylation during neutrophilic differentiation, we performed genome-wide methylation analysis at single CpG sites of CD34+ (t0) and day five progenitor (t5) cells, using the Illumina Infinium HumanMethylation 450 platform. Analysing 412,940 CpGs, we observed a high correlation between all biological replicates and a clear separation of samples from CD34+ and neutrophilic progenitor cells in a principal component analysis (Fig. 3a; Supplementary Fig. S12). As expected, the pattern of DNA methylation changed dramatically during differentiation, resulting in 3,882 hypo- (log2 FC < −0.6) and 2,977 hypermethylated (log2 FC > 0.6) sites (FDR-adjusted *P* value < 0.05) (Fig. 3b). Yet, no CpGs were differentially methylated with statistical significance when ELF-MF-exposed progenitor cells were compared with control conditions (Fig. 3c; Supplementary Fig. S12). These results indicate that, although DNA methylation undergoes major changes during neutrophilic granulopoiesis, ELF-MF exposure does not influence the formation of cell-type-specific DNA methylation patterns.

### ELF-MF exposure affects the variability of epigenetic modifications between replicate experiments

**Figure 4:**
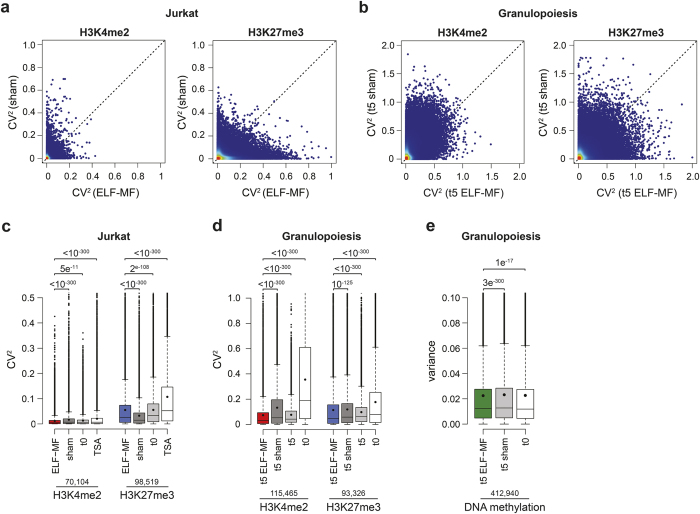
ELF-MF exposure impacts the variability of the epigenetic landscape. The squared coefficient of variation (CV^2^) of ChIP-seq read counts within 500 bp genomic tiles was determined based on the two replicate datasets for H3K4me2 and H3K27me3, generated from non-exposed (t0), ELF**-**MF-exposed (50 Hz sinus, 1 mT, 5′ on/10′ off, 72 h), sham-exposed or Trichostatin A-treated (10 nM, 72 h) Jurkat cells, and from neutrophilic progenitors after five days of *in vitro* differentiation under ELF**-**MF (50 Hz powerline, 1 mT, 5′ on/10′ off) or sham exposure. Linear comparison of CV^2^ values of ELF**-**MF- (x-axis) and sham-exposed (y-axis) Jurkat cells (**a**) and neutrophilic progenitor cells (**b**) for H3K4me2 and H3K27me3 marks. Variability measures of the two replicate ChIP-seq datasets for H3K4me2 and H3K27me3 in Jurkat cells (**c**) and in neutrophilic progenitors (**d**). (**e**) Variance in DNA methylation levels (M value) of the three biological replicates for CD34+ human umbilical cord blood cells (t0), ELF-MF- and sham-exposed neutrophilic progenitors after five days of *in vitro* differentiation. (**c–e**) Box-and-whisker plots illustrate median (lines) and mean (black circles) CV^2^ values with interquartile ranges (boxes), 1.5× interquartile ranges (whiskers) and outliers. *P* values of the Wilcoxon rank sum test and the number of genomic tiles are indicated above and below, respectively.

Although we did not identify consistent alterations of histone modifications or DNA methylation upon ELF-MF exposure both in leukaemic cells and in differentiating neutrophilic cells, we observed a number of genomic loci showing up to 16-fold differential occupancy by H3K4me2 and H3K27me3 or DNA methylation (Figs 1c, 2d and 3c). These differences did not reach statistical significance (FDR-adjusted *P* value < 0.05) due to a considerable variability between the data sets. The analysis of genomic sequencing data involves statistical approaches that detect deterministic events, i.e., responses that are similar in populations of cells and reproducible in replicate experiments. Effects inducing stochastic events, affecting individual cells and populations randomly, would increase the variability between samples and be considered experimental noise. Although the overall correlation of our ChIP-seq data sets was high (Supplementary Figs S3, S4, S9 and S10), a closer examination of the H3K4me2 and H3K27me3 read counts in all tiles revealed exposure condition-dependent effects on the variability of replicate samples, both in Jurkat cells and neutrophilic progenitors (Fig. 4a,b). We reasoned that this difference may reflect an influence of the exposure on the establishment and/or maintenance of H3K4me2 and H3K27me3 modification patterns, resulting in a stochastic perturbation of epigenetic programming. A dramatic replicate variability of H3K4me2 and H3K27me3 marks was also observed in Jurkat cells treated with low-dose TSA, a known epigenetic modulator (Fig. 4c), which corroborates that epigenetic perturbation is indeed detectable at the level of sample variance. ELF-MF exposure, when compared to sham- or non-exposed (t0) conditions, significantly increased the replicate variability of H3K27me3 modifications while reducing the variability of H3K4me2 modifications in Jurkat cell (Fig. 4c). Consistent with the higher epigenetic plasticity of stem and tissue progenitor cells, replicate variabilities of H3K4me2 and H3K27me3 modifications were more pronounced in populations of CD34+ haematopoietic stem cells and neutrophilic progenitors (t5) than in populations of Jurkat cells (Fig. 4c,d). ELF-MF exposure significantly reduced the replicate variability of both H3K4me2 and H3K27me3 modifications in neutrophilic progenitors (Fig. 4d), and the same was apparent in the DNA methylation data (Fig. 4f). Notably, the decrease in replicate variability in ELF-MF-exposed cells correlated well with a more robustly directed differentiation into the neutrophilic lineage (Supplementary Fig. S13). Together, these observations indicate that ELF-MF exposure can affect the robustness of the establishment and/or maintenance of key epigenetic modifications in a global but stochastic manner, particularly in differentiating cell populations.

### The stabilizing effect of ELF-MF on epigenetic modifications partially depends on chromatin state

**Figure 5:**
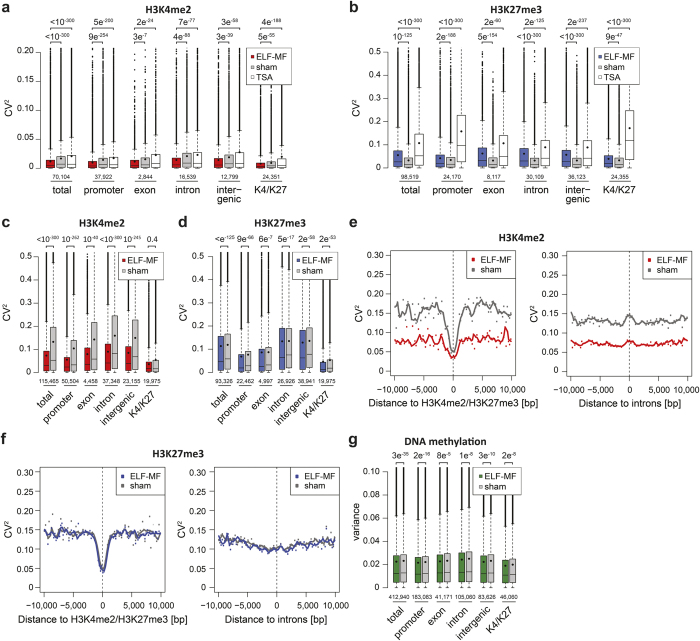
Genomic context-dependent effect of ELF-MF exposure on the robustness of epigenetic modifications. The squared coefficient of variation (CV^2^) of reads in 500 bp tiles of two H3K4me2 and H3K27me3 ChIP-seq replicates and the variance of DNA methylation in the three replicates was analyzed for promoters (±1,000 bp of TSS), exons, introns, intergenic regions (UCSC hg19) or bivalent domains (H3K4me2 and H3K27me3 co-occupancy) in our data set. Box-and-whisker plots illustrate median (lines) and mean (black circles) CV^2^ values with interquartile ranges (boxes), 1.5× interquartile ranges (whiskers) and outliers. *P* values of the Wilcoxon rank sum test and the number of genomic tiles are indicated above and below, respectively. (**a**,**b**) Assessment of the variability of epigenetic modifications in ELF-MF- (50 Hz sinus, 1 mT, 5′ on/10′ off, 72 h) and sham-exposed or TSA-treated (10 nM, 72 h) Jurkat cells with respect to the indicated genomic features. (**c**,**d**) As in (**a**,**b**) but with data from neutrophilic progenitors after five days of *in vitro* differentiation under ELF-MF (50 Hz powerline, 1 mT, 5′ on/10′ off) or sham exposure. (**e**,**f**) Comparison of the mean variabilities of ChIP-seq reads of H3K4me2 and H3K27me3 tiles (CV^2^ values on y-axis) between ELF-MF- and sham-exposed samples of neutrophilic progenitors, plotted as a function of distance to the nearest bivalent domain or intron. (**g**) Variability of three replicates of DNA methylation assessed with respect to genomic features, comparing ELF-MF- and sham-exposed neutrophilic progenitors.

Next, we investigated whether the variability of epigenetic modifications is preferentially associated with certain genomic features or randomly distributed across the genome. We intersected the profiles of histone and DNA modifications with annotated gene promoters, exons, introns and intergenic regions and also correlated them with bivalent chromatin domains identified by co-occupancy of active H3K4me2 and repressive H3K27me3 marks in our ChIP-seq data. In TSA-treated Jurkat cells, variability of H3K4me2 showed little dependency on the genomic context, while H3K27me3 was clearly most variable at gene promoters and in bivalent chromatin (Fig. 5b), consistent with the preferential localisation of histone acetylation at gene promoters and enhancers^42^. ELF-MF exposure, however, affected replicate variability of both H3K4me2 and H3K27me3 modifications irrespective of the genomic context (Fig. 5a,b). In neutrophilic progenitors, H3K4me2 and H3K27me3 modifications generally showed the highest reproducibility at promoters and in bivalent chromatin domains and more pronounced replicate variability in introns and intergenic regions (Fig. 5c). ELF-MF exposure decreased replicate variability of H3K4me2 marks at all genomic locations except in bivalent chromatin domains (Fig. 5c,e; Supplementary Fig. S13). Consistently, active gene promoters, devoid of H3K27me3, were significantly more stabilized by exposure than bivalent promoters, co-occupied by H3K4me2 and H3K27me3 modifications (Supplementary Fig. S13). ELF-MF exposure also reduced the variability of H3K27me3 modifications, although the reduction was less pronounced and without much preference for any of the genomic sites assessed (Fig. 5d,f; Supplementary Fig. S13). The variability of DNA methylation in neutrophilic progenitors was also significantly decreased upon ELF-MF exposure, although without much preference for a particular genomic context (Fig. 5g). These results indicate that ELF-MF affects the robustness of epigenetic marks in a partially genomic context-dependent manner, with the most distinctive feature being the H3K4me2 modification in active and bivalent gene promoters of differentiating cells.

**Figure 6:**
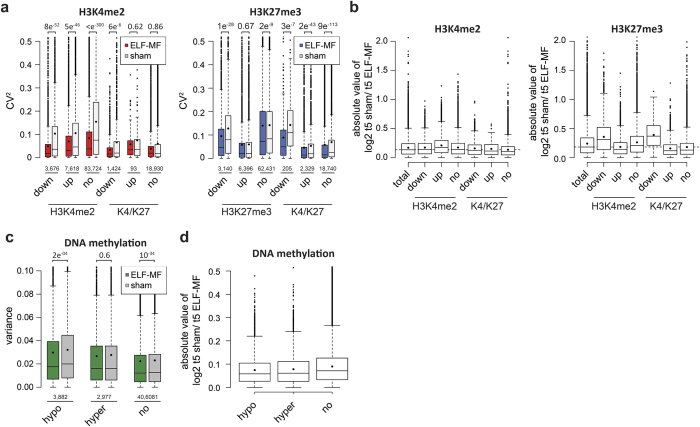
The chromatin state defines the stability of epigenetic features under ELF-MF exposure. Variability of epigenetic marks in sham- and ELF-MF-exposed neutrophilic progenitors was intersected with tiles/sites that change significantly (FDR-adjusted *P* < 0.05) epigenetic modifications during differentiation of CD34+ cells to day five progenitors. (**a**) Median (lines) and mean (black circles) CV^2^ values with interquartile ranges (boxes), 1.5× interquartile ranges (whiskers) and outliers, categorized according to tiles enriched in H3K4me2, H3K27me3 or both that either significantly change (up, log2-fold change >0.6; down, log2-fold change <−0.6) or remain stable (no) during differentiation. (**b**) As in (**a**) but for differential enrichments of histone modifications between ELF-MF- and sham-exposed samples. (**c**,**d**) Variability of DNA methylation of sham- and ELF-MF-exposed neutrophilic progenitors, intersected with CpGs significantly changing methylation (FDR-adjusted *P* < 0.05; hypo: log2-fold change <−0.6; hyper: log2-fold change >0.6) or not (no) during neutrophilic differentiation. Shown are median (lines) and mean (black circles) with interquartile ranges (boxes), 1.5× interquartile ranges (whiskers) and outliers of variance (**c**) and log2-fold changes between ELF-MF- and sham-exposed samples (**d**). (**a**,**c**) *P* values above indicate the statistical significance level by the Wilcoxon rank sum test and the number of included genomic tiles or CpG sites is shown below.

Bivalent chromatin is associated with transcriptionally poised states and often present at regulatory elements of developmental genes that will be activated or repressed in the course of cell differentiation^30^,^43^. As bivalent chromatin is highly dynamic but seemingly less variable and more protected from ELF-MF impact in differentiating neutrophilic progenitors, we investigated whether the robustness of H3K4me2 and H3K27me3 marks correlates with differentiation-associated changes in chromatin status. We identified genomic tiles changing H3K4me2 or H3K27me3 modification (FDR-adjusted *P* value < 0.05) during neutrophilic differentiation and categorized them into upregulated (log2 FC > +0.6), downregulated (log2 FC < −0.6) or unchanged for either of the histone modifications. Then, we examined the representation of the three categories of tiles with differentiation-associated changes in tiles showing H3K4me2 and/or H3K27me3 occupancy in neutrophilic progenitors (Fig. 6a). The analysis of these categories confirmed that ELF-MF exposure generally reduced H3K4me2 variability in active (H3K4me2) but not in bivalent chromatin (H3K4me2 and H3K27me3), and this effect was independent of the differentiation dynamics in either of the modifications (Fig. 6a). By contrast, ELF-MF exposure reduced the variability of H3K27me3 modification with a clear dependency on chromatin dynamics; the effect was most pronounced in tiles losing H3K27me3 during differentiation, regardless of the status of bivalency (Fig. 6a).

We then examined the relationship between the differentiation dynamics of the histone modifications and the ELF-MF effect on their relative enrichment (Fig. 6b). This revealed a notable ELF-MF effect on H3K4me2 occupancy in tiles representing active chromatin (H3K4me2 only) that gained H3K4me2 during differentiation, as well as an effect on H3K27me3 occupancy in tiles that lost the modification during differentiation. To address whether ELF-MF exposure affects DNA methylation in a similar way, we analyzed replicate variability of DNA methylation data from neutrophilic progenitors in CpG sites that are significantly hypo- (log2 FC < −0.6, FDR-adjusted *P* value < 0.05) or hyper-methylated (log2 FC > +0.6, FDR-adjusted *P* value < 0.05), or unchanged in the course of differentiation. Consistent with the data on H3K27me3, ELF-MF exposure was associated with a significantly reduced variability of CpG methylation at sites losing 5mC during differentiation but had no effect at sites gaining 5mC. We also observed that the impact of ELF-MF on DNA methylation levels in neutrophilic progenitors was less pronounced at CpGs that change methylation during differentiation than at sites that show no changes (Fig. 6d). These observations suggest that ELF-MF exposure stabilizes epigenetic modifications in regions marked by the active histone mark H3K4me2. This stabilizing effect is most pronounced in chromatin that changes from a repressive to an active state during differentiation, i.e., that loses H3K27me3 and/or undergoes DNA demethylation while consolidating H3K4me2 marks.

## Discussion

Epidemiological studies have associated the exposure to ELF-MF with an increased risk of childhood leukaemia but underlying biological mechanisms have remained elusive. The concept of cancer promotion through DNA damage-induced genetic mutation is widely accepted for ionizing radiation but appears, on the basis of energetic considerations, not applicable to ELF-MF. We reasoned that ELF-MF exposure may affect the epigenome rather than the genome, thereby promoting cancerous changes in cell identity and behaviour. To explore this possibility, we examined if ELF-MF exposure influences the stability and programming of key epigenetic modifications in leukaemic cells and in differentiating haematopoietic stem cells in a way that may explain enhanced leukaemogenesis.

The epigenome is well-known to be susceptible to environmental influences of all kinds, including the exposure to non-mutagenic carcinogens^22^,^23^,^35^. We showed here that the treatment of leukaemic Jurkat cells with a very low, sub-toxic dose of TSA destabilizes the patterns of key epigenetic modifications (H3K4me2 and H3K27me3). While the TSA treatment did not affect cell survival and phenotype in any notable way, epigenetic destabilization was detectable by genome-wide profiling, documenting the feasibility and sensitivity of our approach to measure subtle aberrations in histone modifications. Yet, the evaluation of the impact of ELF-MF exposure on the profile of these marks did not reveal any specific and reproducible exposure-dependent alterations in Jurkat cells. We therefore conclude that in the well-controlled experimental setup of this study, ELF-MF exposure does not cause perturbations of the epigenetic landscape in a leukaemic cell line. Likewise, we observed no statistically significant alterations of epigenetic modifications in neutrophilic progenitors after five days of *in vitro* differentiation under ELF-MF exposure. Neither H3K4me2, nor H3K27me3, nor DNA methylation marks were influenced by the exposure, although extensive differentiation-associated changes in these modifications were clearly evident. The absence of an impact of the ELF-MF on CpG methylation levels and patterning during neutrophilic granulopoiesis is in contrast to previous work documenting changes in global cytosine methylation of a murine spermatocytes-derived cell line when exposed to a comparable ELF-MF (50 Hz, 1 or 3 mT, intermitted, 72 h)^31^. This discrepancy could be explained by cell-type-specific susceptibilities of the DNA methylation system to ELF-MF exposure or by a difference in permissiveness of the culture systems for the establishment of epigenetic variation in subpopulations of cells. Our *in vitro* differentiation system was tightly controlled by the growth factor G-CSF and cytokines, strongly favouring growth and development of cells of the neutrophilic lineage while restricting the establishment of other cell-types. Minor exposure-dependent, transient differences in cell cycle progression and apoptosis early in the differentiating cell populations may indeed reflect an underlying positive selection for neutrophilic cells, which supported the formation of homogenous populations of promyelocytes and myelocytes, irrespective of the ELF-MF exposure. So, selection may have masked epigenetic divergence in our experiments, which may have been picked up more easily by the less stringent conditions in the experiments with spermatocyte-derived cells. ELF-MF exposure of these cells might have triggered spontaneous differentiation responses that were tolerated in the culture and, ultimately, gave rise to detectable epigenetic change. Notably, an enhancement of differentiation upon ELF-MF exposure was reported in neuronal cells^24^,^27^,^44^, where it correlated with a gain of H3K9 acetylation at specific neuronal genes, promoting their activation^45^.

Our data thus suggests that ELF-MF exposure has no specific and reproducible effect on key epigenetic modifications in leukaemic cells and in differentiating haematopoietic cells. We cannot rule out potential effects on histone modifications that were not studied here or would become evident under different experimental conditions, e.g. under prolonged duration or a different mode of ELF-MF exposure or with different cell lines or cell differentiation protocols. A closer examination of the data, however, revealed a potential impact of ELF-MF exposure on the robustness of the epigenetic programming. ELF-MF exposure was generally associated with a reduced variability in ChIP-seq and DNA methylation data between replicate samples, the exception being H3K27me3 in Jurkat cells, showing increased variability upon ELF-MF exposure. Notably, the treatment of Jurkat cells with TSA – a known epigenetic modulator – generated similar effects on replicate variability for both histone modifications, suggesting that the variance in replicate data sets can be a measure for epigenetic robustness, which appears to be perturbed in a stochastic manner in the case of ELF-MF exposure. The impact of ELF-MF exposure on the reproducibility of histone and DNA modification patterns is likely to depend on cell-type-specific epigenetic states and plasticity, as well as on differential sensitivities to ELF-MFs^46^,^47^,^48^. For instance, it was reported that chromatin conformation changes following low dose ELF-MF exposure of human lymphocytes depend on the initial chromatin state^49^. Investigating the viscosity of chromatin, the authors found that relaxed chromatin becomes more condensed and initially compact chromatin more open upon ELF-MF exposure, i.e., that chromatin perturbance by ELF-MFs is context-dependent. Our *in vitro* differentiation starts with an epigenetically dynamic and heterogeneous stem cell population^50^,^51^,^52^, as indicated by a high replicate variability for H3K4me2 and H3K27me3 modifications in CD34+ cells. ELF-MF exposure then appears to exert a homogenizing effect on the initially variable chromatin states in the early differentiating cell population, hence reducing the replicate variability at later time-points in differentiation. We found that ELF-MF exposure increases the robustness of histone modifications as well as DNA methylation, particularly in genomic regions marked by H3K4me2, which lose H3K27me3 and/or DNA methylation in the course of differentiation. As H3K4me2 and unmethylated CpGs are associated with transcriptionally active chromatin, allowing for binding of transcription factors, the locus-specific pattern of reduced replicate variability upon ELF-MF exposure coincides with sites of transcriptional activity and/or chromatin opening during differentiation. These results therefore suggest that open and active chromatin is more affected by ELF-MF exposure than condensed chromatin.

Notably, to monitor the status of our cell cultures and to assess the comparability of the genomic data produced, we systematically measured a variety of cell growth parameters throughout all experiments. Previous studies reported ELF-MF exposure-dependent alterations in cell proliferation, cell cycle progression and apoptosis in various cancerous and non-cancerous cells^20^,^33^,^39^,^53^,^54^. Upon ELF-MF exposure, we did not observe any significant effects on cell proliferation in Jurkat cells and during granulopoiesis, nor on apoptosis or cell cycle progression in Jurkat cells. During granulopoiesis, we found small differences in cell cycle progression concomitant with increased apoptosis in ELF-MF-exposed cultures at day four. This may have had a synchronizing effect on exposed cell populations and thereby contributed to the reduced replicate variability of epigenetic features observed for these cultures.

In conclusion, we report that ELF-MF exposure has no significant effect in a deterministic manner on the epigenetic landscapes of leukaemic and differentiating haematopoietic cells. However, our data indicate that ELF-MF exposure may influence the robustness of histone modification and DNA methylation patterning in the course of the global chromatin reorganization associated with neutrophilic differentiation. This, however, did not affect notably the overall dynamics and efficiency of granulopoiesis.

## Methods

### Cell culture and neutrophilic differentiation

Culturing was routinely performed at 37 °C in a humidified atmosphere with 5% CO_2_. Jurkat cells (acute T cell leukaemia) were cultured in RPMI-1640 medium supplemented with 2 mM L-glutamine, 1 mM Na-pyruvate, 10% FCS and 0.6x penicillin/streptomycin (Sigma-Aldrich). Fresh medium was supplied every 48 h. For ELF-MF exposure experiments, cells were seeded at a density of 10^5^ cells/mL and grown for the indicated time without addition of fresh medium. As positive control, cells were treated with 10 nM trichostatin A (TSA). CD34-positive cells isolated from human cord blood of mixed donors were obtained from AllCells (Alabama, United States) and propagated in Stemline II medium expansion medium (Stemcell technology) supplemented with 100 ng/mL thrombopoietin, 100 ng/mL stem cell factor, 10 ng/mL Flt3-ligand (Peprotech), 5000 U/mL Penicillin and 5 mg/mL Streptomycin (Sigma-Aldrich) for four days^55^. For the neutrophilic differentiation, expanded CD34+ cord blood cells were seeded at cell densities of 10^5^ cells/mL in Stemline II medium supplemented with 100 ng/mL stem cell factor, 10 ng/mL Flt3-ligand, 100 ng/mL G-CSF (Peprotech), 5000 U/mL Penicillin and 5 mg/mL Streptomycin (Sigma-Aldrich) and cultured for 10 days. Every second day, fresh cell culture medium was added (1:2 dilution).

### Exposure to ELF-MF

The exposure system sXcELF (IT’IS, Zurich, Switzerland) allows for a well-controlled exposure with ELF-MF (Supplementary Fig. S1)^56^. Leukaemic cell lines were exposed with a sinusoidal ELF-MF (50 Hz, 1 mT, 5′ on/10′ off) or sham for approximately 3.5 cell cycles (72 h). The electric field caused by the coil was shielded and the magnetic field uniformity was better than 1% (SD). Each coil was placed inside a μ-metallic box and the boxes placed side by side in the same incubator to decouple the coils, to shield the electric and magnetic fields generated by the incubator and to ensure the same environmental conditions (temperature, humidity and CO_2_). The field attenuation between the coils was at least a factor of 150, i.e., sham exposure was anywhere less than 7 μT compared to the uniform exposure of 1 mT. The exposure system meets the general criteria established in [Recommended Minimal Requirements and Development Guidelines for Exposure Setup of Bio-Experiments Addressing the Health Risk Concern of Wireless Communications]. Intermittent exposure was chosen as it reflects better *in situ* scenarios than continuous exposures; it has also been demonstrated to enhance the response, i.e., 5′ on/10′ off cycles showed the largest ELF-MF response on comet assay tail factors. Additional controls were done in parallel, either in a μ-metal shielded compartment inside the exposure incubator or in different incubator. Randomized assignment of ELF-MF and sham exposure conditions to the two chambers allows experiments blinded for the observer. Continuous temperature monitoring confirmed that the temperature difference between the exposure chambers was less than 0.1 °C.

### Measurement of cell cycle, apoptosis and identification of cell differentiation state by flow cytometry

Cell cycle profiles were analyzed by propidium iodide staining as described in detail elsewhere^18^. The number of apoptotic cells in the population was estimated by the Annexin-V Alexa488/PI kit (Invitrogen) according to the provider’s recommendations. Maturation status of the differentiating neutrophilic cell population was analyzed by immuno-detection of cell surface markers. The following combination of antibodies were used: PE mouse anti-human CD16b, APC mouse anti-human CD34, APC-Cy7 mouse anti-human CD11b, PE-Cy7 mouse anti-human CD14 (all from BD Biosciences) and FITC-anti-Annexin V (Invitrogen). Neutrophilic differentiation stages were identified by gating on subpopulations according to the expression of surface markers (Supplementary Fig. S6)^57^: CD34+ cells (CD34+, CD11b−, CD16b−), promyelocytes (CD34−, CD11b−, CD16b−), myelocytes (CD34−, CD11b+, CD16b−) and metamyelocytes (CD36−, CD11b+, CD16b+). Monocytes were identified according to the expression of CD14+. All samples were measured with a FACS cytometer (BD Biosciences) and analyzed by the FlowJo software. Data were statistically analyzed by χ^2^ test for each replicate as well as pairwise comparison by Student’s *t*-test in GraphPad Prism. Full methodical details are given in the Supplementary information online.

### Base resolution DNA methylation analysis by Illumina Infinium HumanMethylation 450 array

Genomic DNA was extracted from frozen cell pellets by QIAamp DNA mini kit (Qiagen) according to the manufacturer’s instructions, including an RNase-treatment step. 500 ng of genomic DNA was bisulfide-converted using the EZ-96 DNA Methylation Kit (Zymo Research Corporation). Genome-wide assessment of DNA methylation was done on Illumina Infinium HumanMethylation 450 Beadchip arrays, interrogating methylation at 485,577 sites^58^. Raw signal intensities were extracted by the Illumina GenomeStudio software and imported into R as a methylumi object in the methylumi package. Data normalization was performed applying the dasen method as described previously^59^. Briefly, probe-level signals for individual CpG sites were subjected to background adjustment, followed by quantile normalization of both typeI and typeII probes separately. Probes for CpG sites with signal intensities not significantly different (*P* < 0.05) from background measurements in any data sets or mapping to regions with known germline polymorphisms, to multiple genomic loci^60^, or to sex chromosomes were removed, yielding a total of 412,940 CpG after filtering. All computational and statistical analyses were performed in R and Bioconductor^61^. All analyses for differential methylation were performed on M-values (M = log2 (methylated/unmethylated) as recommended^62^. Empirical Bayes methodology utilizing a moderated *t*-statistic available in limma was used to test for significant differences between the groups. False-discovery rate (FDR)-adjusted *P* values for multiple comparisons were calculated by the Benjamini and Hochberg approach. Differentially methylated CpGs were defined as those with both a false discovery rate (FDR)-adjusted *P* value < 0.05 and log2-fold change >0.6. Variance was calculated based on M-values^62^.

### Whole-genome analysis of histone modifications

The ChIP protocol using ChIP-validated antibodies for the H3K4me2 and H3K27me3 histone modifications (Millipore) of 20–30 μg chromatin was performed as described previously^63^ and in more detail in the Supplementary information online. Single-end 50 bp read sequencing of ChIP was performed at the Quantitative Genomics Facility in Basel and at the Genome Technology Access Center (GTAC) in St. Louis (Missouri, USA), using standard protocols for library generation for the Illumina HiSeq platform. To maintain the blinding of ELF-MF exposure conditions during ChIP-seq and bioinformatics analyses, sample pooling was guided by the IT’IS Foundation. In total, 16 libraries were sequenced from H3K4me2 and H3K27me3 ChIP samples of Jurkat cells: 2 sample pools of 3 independent exposure replicates for each condition (before exposure, sham, ELF-MF exposure and TSA treatment) and both histone modifications, resulting in around 200 Mio reads for the exposure conditions and 50 Mio reads for the TSA-treated samples. For ChIP-seq of primary cells during neutrophilic differentiation, 14 libraries were generated from H3K4me2 and H3K27me3 ChIP samples: one independent exposure replicate as well as one pool of two independent exposure replicates for each exposure condition (ELF-MF and sham) for CD34+ cells and neutrophilic progenitors at day 5. For the control differentiation, ChIPs of two independent replicates of progenitor cells were pooled. Additionally, two input controls were included, one each for CD34+ and progenitor cells.

The analysis of ChIP-seq data was performed at the scientific computing core facility (sciCORE) of the University of Basel. For each library, sequence reads were aligned to the human reference genome assembly (hg19) using the Sequence Mapping and Alignment Tool (SMALT v 0.6.2). High quality alignments (bamtools filter-mapQuality “>30”) were extracted. Center positions of ChIPped DNA fragments were approximated based on average fragment lengths and orientations of read alignments (http://ccg.vital-it.ch/chipseq/chip_center.php). Fragment center-positions of each library were used to call genomic domains for H3K4me2 or H3K27me3 with increased read densities applying the program “chippart” (http://ccg.vital-it.ch/chipseq/). Global domain sets were assembled by combining domains less than 1 kb apart with the merge function of bedtools and being present in at least one of the samples. Merged domain sets were subdivided into tiles of uniform lengths of 500 and 1,000 bp. After random downsampling of high quality alignments (51 Mio and 26 Mio for Jurkat and HSC, respectively), the number of reads mapping within tiles (fragment center-positions) were extracted for each library. Tiles were further filtered for read counts above an arbitrary threshold above background (50 reads for H3K4me2, 30 for H3K27me3 per 500 bp tile and 100 reads for H3K4me2, 60 for H3K27me3 per 1,000 bp tile length) in at least one sample. In the differentiation experiment, an additional filtering criteria of 7 read counts above the corresponding input was applied. Read counts within genomic tiles were tested for differences between groups of samples applying a generalized linear model (GLM) likelihood ratio test as implemented in the EdgeR package, originally developed for differential gene expression data^64^. In brief, the dispersion parameter of the negative binomial model was estimated for each genomic interval. Tables with read counts resulting from the merging, normalization and filtering were used and the log2-fold changes and *P* values of the GLM likelihood ratio test were computed under the null hypothesis that the fitted coefficients of negative binomial GLMs of the compared groups are equal. Domains (500 bp and 1,000 bp) with significant alterations were defined as those with both a false discovery rate (FDR)-adjusted *P* value < 0.05 and log2-fold change >±0.6. If overlaying 500 and 1,000 bp tiles had significant alteration the 500 bp tile was selected.

The integrative genomics viewer was used to visualize ChIP-seq reads^65^,^66^, deposited in NCBI’s Gene Expression Omnibus^67^. Coefficient of variation and the principal component coefficients were calculated based on the normalized read counts from EdgeR comparisons of 500 bp domain of ChIP-seq datasets and analyzed by R/Bioconductor. Intersections between H3K4me2 and H3K27me3 ChIP-seq data in 500 bp tiles indicate bivalent domains. Promoter, exons, introns, intergenic regions and transcription start sites were defined in the Bioconductor package TxDb.Hsapiens.UCSC.hg19.knownGene and analyzed by R/Bioconductor^61^.

### Data Availability

Geo reviewer links: https://www.ncbi.nlm.nih.gov/geo/query/acc.cgi?acc=GSE85392, https://www.ncbi.nlm.nih.gov/geo/query/acc.cgi?acc=GSE85562 and https://www.ncbi.nlm.nih.gov/geo/query/acc.cgi?acc=GSE85601.

## Additional Information

**Accession codes:** Raw and processed data of ChIP-seq and DNA methylation analyses have been deposited in NCBI’s Gene Expression Omnibus (GEO) database and are accessible through the GEO accession numbers GSE85392 (DNA methylation), GSE85562 and GSE85601 (histone modifications).

**How to cite this article:** Manser, M. *et al*. ELF-MF exposure affects the robustness of epigenetic programming during granulopoiesis. *Sci. Rep.* **7**, 43345; doi: 10.1038/srep43345 (2017).

**Publisher's note:** Springer Nature remains neutral with regard to jurisdictional claims in published maps and institutional affiliations.

## Supplementary Material

Supplementary Information
