# Supplementary material for: ELF-MF exposure affects the robustness of epigenetic programming during granulopoiesis
Source: Sci Rep. 2017 Mar 7;7:43345. doi: 10.1038/srep43345 (PMC5339735; doi:10.1038/srep43345)
Supplement: Supplementary Information [file srep43345-s1.pdf]

Supplementary Information

ELF-MF exposure affects the robustness of epigenetic programming during granulopoiesis

Melissa Manser[1], Mohamad R. Abdul Sater[2,3,#], Christoph D. Schmid[2,3], Faiza Noreen[1], Manuel Murbach[4], Niels Kuster[4,5], David Schuermann[1,*], Primo Schär[1]

[1] Department of Biomedicine, University of Basel, Mattenstrasse 28, Basel CH-4058, Switzerland

[2] Swiss Tropical and Public Health Institute, Socinstrasse 57, Basel CH-4002, Switzerland

[3] University of Basel, Petersplatz 1, Basel, CH-4001, Switzerland

[4] IT'IS Foundation, Zeughausstrasse 43, Zürich, CH-8004, Switzerland

[5] Swiss Federal Institute of Technology (ETH), Zürich, CH-8006, Switzerland

[#]Present address: Department of Immunology and Infectious Disease, Harvard T.H. Chan School of Public Health, Boston, MA 02115, United States of America

[*]Correspondence and requests for materials should be addressed to D.S. (david.schuermann@unibas.ch)

Supplementary Figures S1—S13

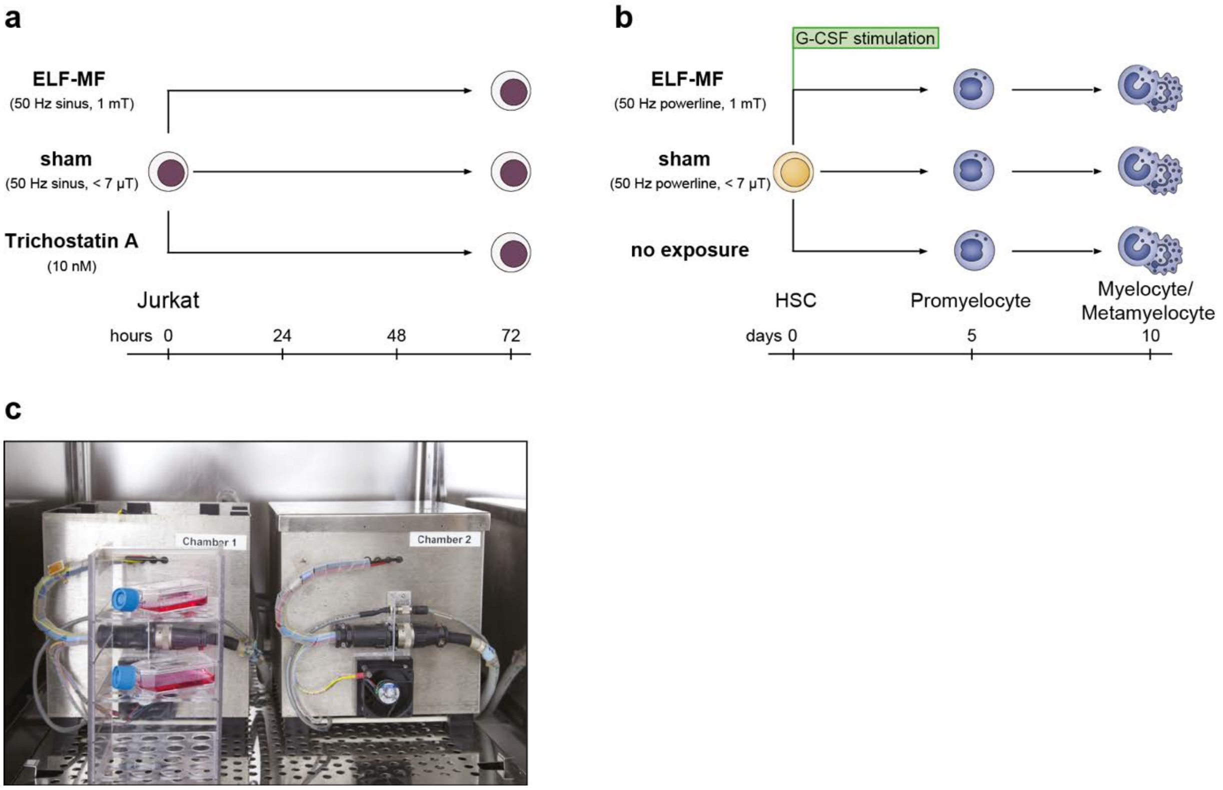

Supplementary Figure S1. Schematic overview of ELF-MF exposure experiments. (**a**) Blinded for the operator, the leukaemic cell line Jurkat was exposed to ELF-MF (50 Hz sinus, 1 mT, 5’ on/10’ off) and sham for 72 h, or treated with 10 nM trichostatin A for 72 h. (**b**) After the expansion of CD34+ human cord blood cells for 4 days, the haematopoietic stem cells (HSC) population was split into three experimental groups before initiating the *in vitro* differentiation into neutrophilic lineage for 10 days by the addition of G-CSF. Blinded for the experimenter, ELF-MF (50 Hz powerline, 1 mT, 5’ on/10’ off) or sham exposure was carried out throughout the differentiation. Control differentiations without ELF-MF exposure were done either in a µ-metal shielded box inside the exposure incubator or in an independent incubator. (**c**) sXcELF exposure system provided and serviced by the IT’IS foundation (http://www.itis.ethz.ch/services/exposure-systems/in-vitro-sxc/sxcelf/).

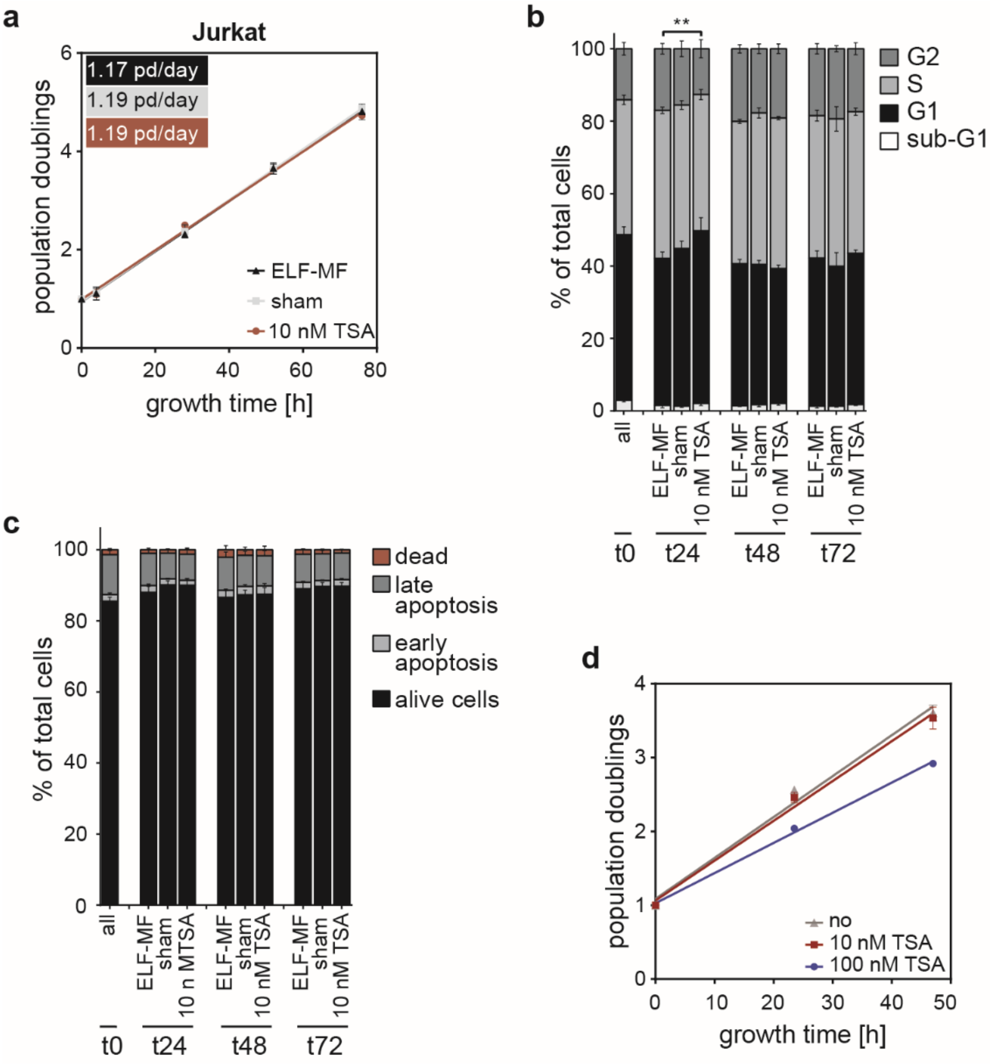

Supplementary Figure S2. ELF-MF exposure does not alter proliferation and survival of Jurkat cells. Cells were ELF-MF-exposed (50 Hz sinus, 1 mT, 5' on/10' off), sham-exposed or treated with 10 nM Trichostatin A (TSA) for 72 h. (**a**) Starting with $1x10^5$ cells/mL, the proliferation was monitored by cell counting at the indicated time-points. Average population doublings (pd) of independent biological replicates (n=6) as a function of time were calculated and statistically analysed by ANOVA and Student's *t*-test (* $P < 0.05$). Error bars indicate SEM. (**b**) Cell cycle profiles were assessed by flow cytometry, before (t0) and after exposure to ELF-MF, sham or TSA at the indicated time-points. Data represent the mean proportion of cells in the different cell cycle phases with SEM (n=5). (**c**) Analysis of apoptosis by flow cytometry of cell populations at the indicated time-points of exposure. Annexin-V/PI staining was used to discriminate living, apoptotic and dead/necrotic cells. Data represent mean percentage of cells in different states of cell viability with SEM (n=6). Flow cytometry data were analysed by FlowJo software and statistically analysed by χ^2 test for each replica and Student's *t*-test (* $P < 0.05$). (**d**) Average population doublings of Jurkat cells treated with 10 nM or 100 nM Trichostatin A (TSA) for 48 h compared to untreated cells. Indicated are error bars and SEM of two biological replicates.

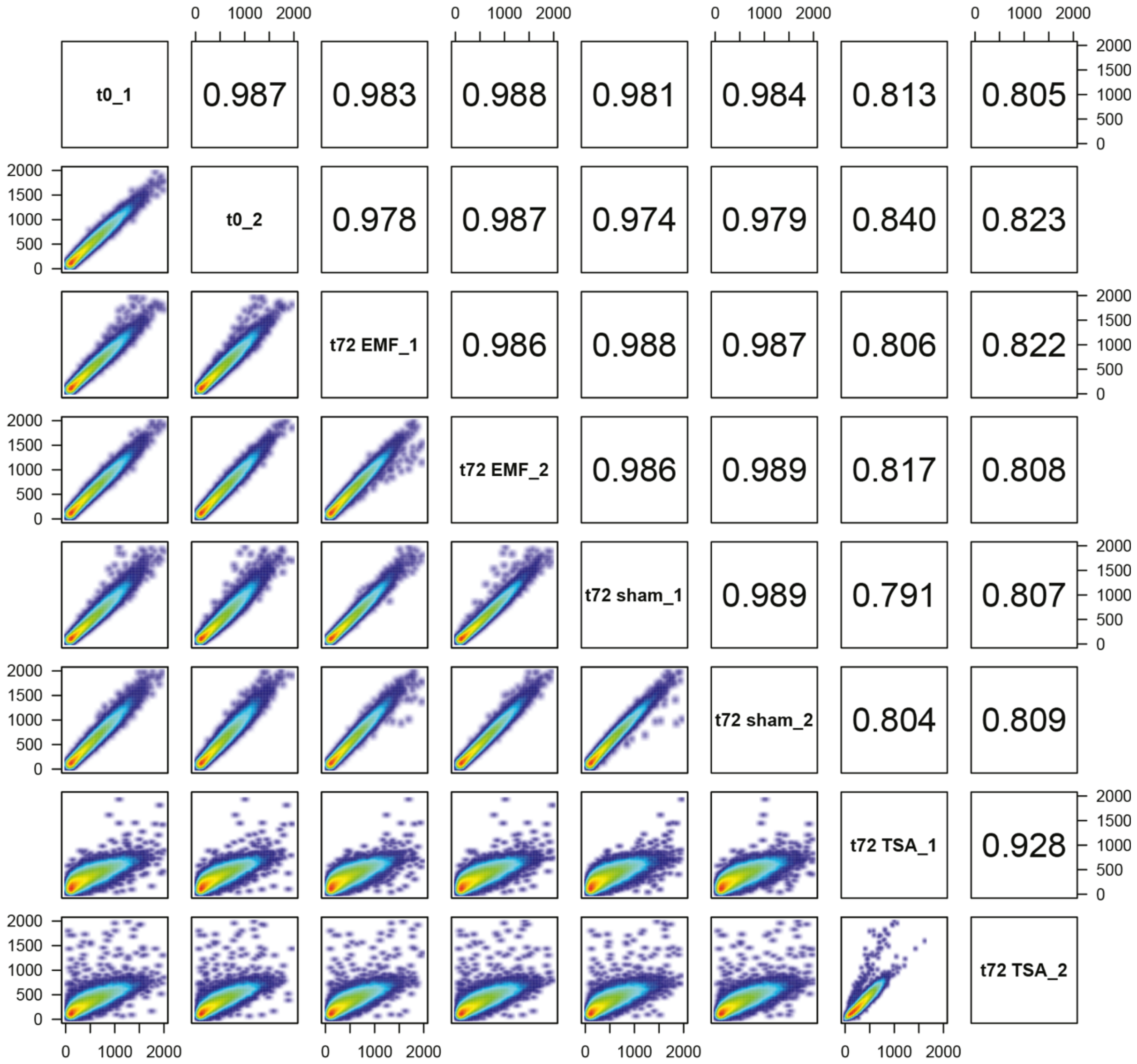

Supplementary Figure S3: Correlation between H3K4me2 ChIP-seq data from Jurkat cells. Global profiles of H3K4me2 histone modification of Jurkat cells prior to treatment (t0), exposed to ELF-MF (t72 EMF; 50 Hz sinus, 1 mT, 5' on/10' off) and sham, or treated with 10 nM trichostatin A (TSA) for 72 h were generated by ChIP-sequencing. For each condition, two ChIP-seq replicates were generated by pooling three biological replicates each. The correlation of ChIP-seq reads in 500 bp genomic tiles between all H3K4me2 ChIP-seq samples is illustrated by density plots (lower left) and correlation coefficients (R values; upper right).

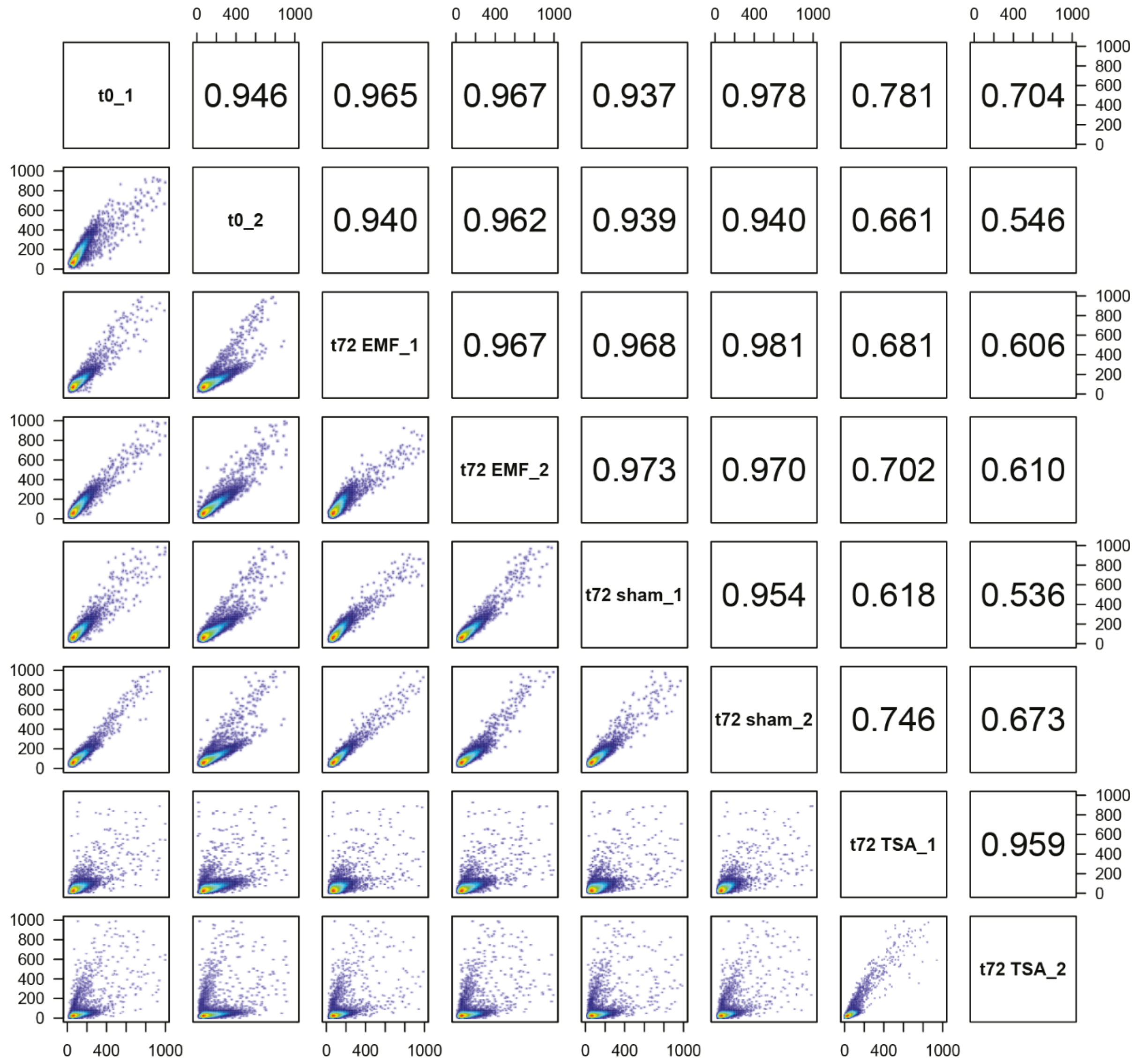

Supplementary Figure S4. Correlation between H3K27me3 ChIP-seq data from Jurkat cells. Global profiles of H3K27me3 histone modification of Jurkat cells prior to treatment (t0), exposed to ELF-MF (t72 EMF; 50 Hz sinus, 1 mT, 5' on/10' off) and sham, or treated with 10 nM trichostatin A (TSA) for 72 h were generated by ChIP-sequencing. For each condition, two ChIP-seq replicates were generated by pooling three biological replicates each. The correlation of ChIP-seq reads in 500 bp genomic tiles between all H3K27me3 Chip-seq samples is illustrated by density plots (lower left) and correlation coefficients (R values; upper right).

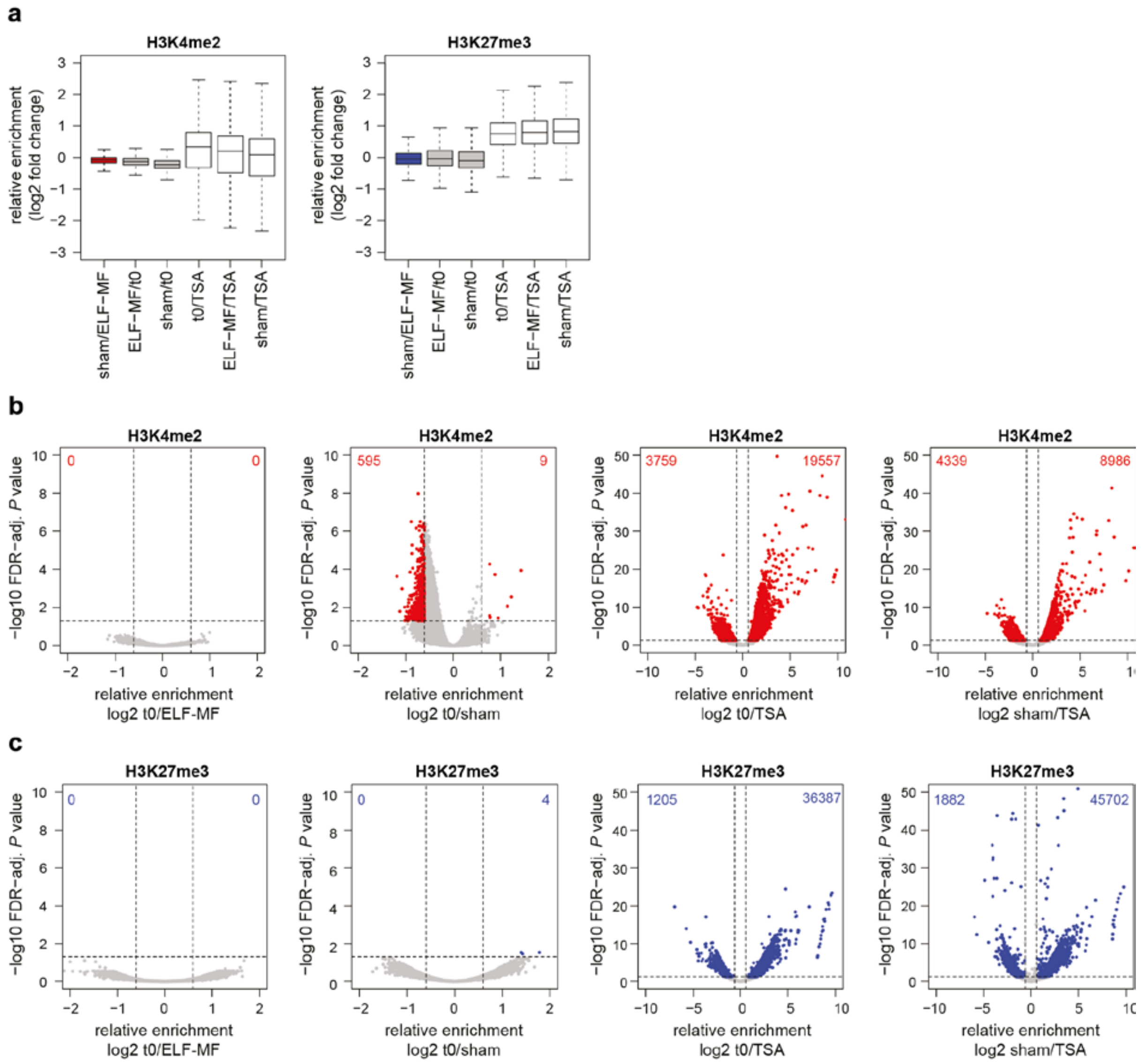

Supplementary Figure S5. Differences in the enrichment of H3K4me2 and H3K27me3 in Jurkat ChIP-seq samples. Jurkat cells were ELF-MF- (50 Hz sinus, 1 mT, 5’ on/10’ off), sham-exposed, or treated with 10 nM Trichostatin A (TSA) for 72 h. Profiles of histone H3K4me2 and H3K27me3 modification were generated by ChIP-seq and alignment to the hg19 genome alignment. Two ChIP-seq replicates (pools of three biological replicates) were statistically analysed. Pairwise comparison of histone modification profiles of cells before exposure (t0), exposed to ELF-MF or sham, or treated with TSA. (**a**) Box-and-whisker plots illustrate the median values (line) of log2 fold changes of H3K4me2 or H3K27me3 ChIP-seq read counts within 500 bp tiles with interquartile ranges (boxes), 1.5× interquartile ranges (whiskers) and outliers. Differences in relative enrichments of H3K4me2 (**b**) and H3K27me3 (**c**) are shown as log2-fold change (FC) (x-axis) and plotted against the false discovery rate (FDR)-adjusted *P* value (calculated by likelihood ratio test) on the y-axis. Statistically significant tiles (FC > ±0.6, FDR-adjusted *P* < 0.05) are highlighted in red (H3K4me2) or blue (H3K27me3).

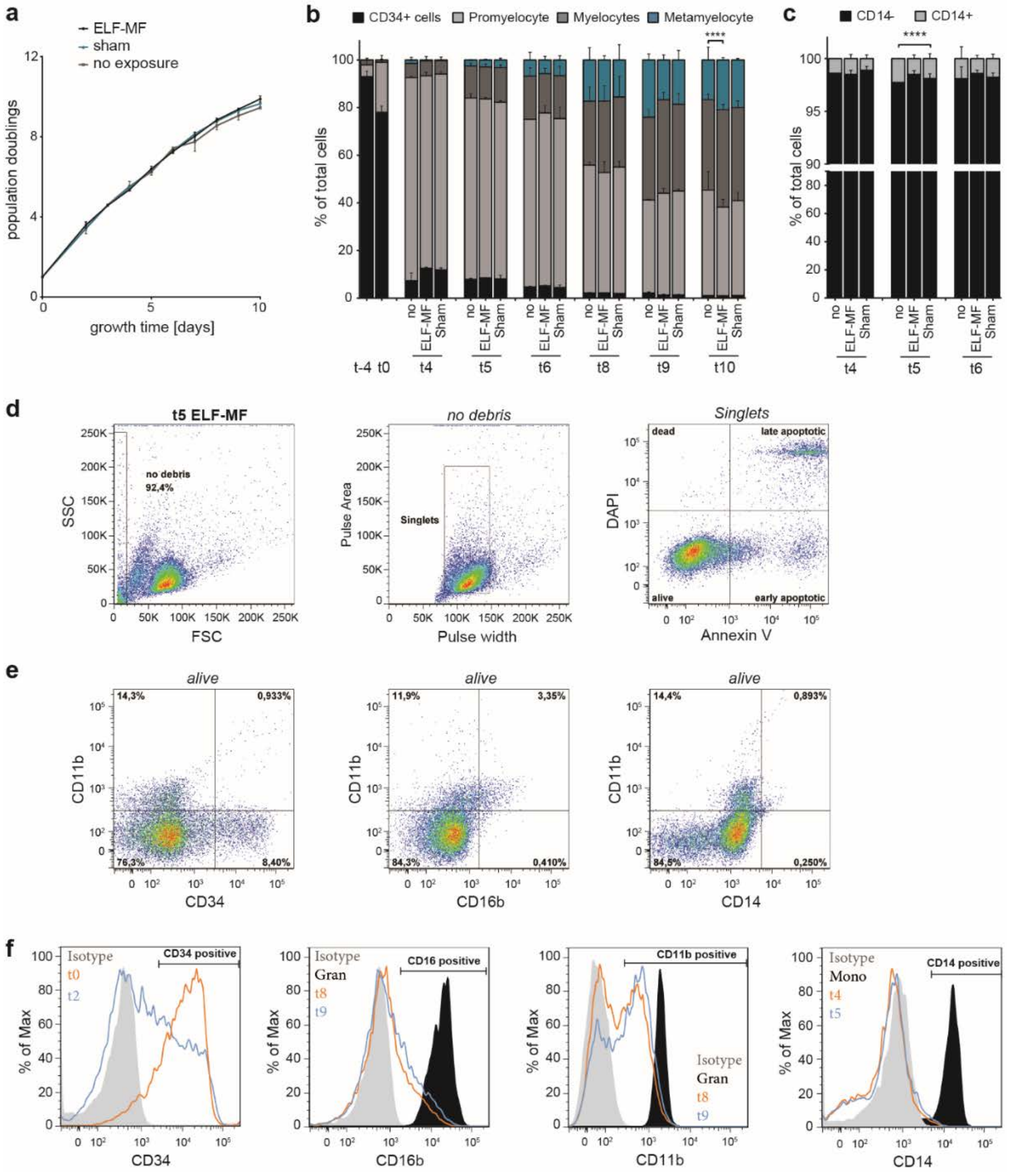

Supplementary Figure S6: ELF-MF exposure has no impact on lineage commitment. Exposed either to ELF-MF (50 Hz powerline signal, 1 mT, 5' on/10' off) and sham or non-exposed negative control, CD34+ cord blood cells were differentiated *in vitro* into neutrophilic lineage for 10 days. (**a**) Cell proliferation of the differentiating cell population monitored daily by counting. Average of population doublings of three independent biological replicates are shown as function of time, statistically analysed by ANOVA and Student's *t*-test. (**b**) Living cells were analysed by flow cytometry at the time-points indicated for neutrophilic differentiation stages, using the stem cell marker CD34 and the two neutrophilic markers CD11b and CD16b. Using the FlowJo software, the expression of these markers was used to discriminate between CD34+ cells (CD34+, CD11b-, CD16b-), promyelocytes (CD34-, CD11b-, CD16b-), myelocytes (CD34-, CD11b+, CD16b-) and metamyelocytes/neutrophils (CD34-, CD11b+, CD16b+). (**c**) Flow cytometric analysis of the population for cells differentiating into granulocytic or monocytic lineage after 4 to 6 days, characterized by CD14 expression. (**b, c**) Shown are the means of three independent replicates (two for the negative control) with SEM, statistically analysed by χ^2 test for each replica and pairwise comparison by Student's *t*-test ($P < 0.05$). (**d, e**) Gating strategy combining the analysis of apoptosis and discrimination of neutrophilic differentiation stages in the alive cell fraction. (**d**) To obtain intact and single (singlet) cells, cell debris and clusters were excluded by gating on forward scatter (FSC)/side scatter (SSC) and on pulse-width, respectively. Single cells were separated into alive, early apoptotic, late apoptotic and dead/necrotic cells according to Annexin-V and DAPI staining. (**e**) Alive cells (Annexin-V negative/ DAPI negative) were then analysed for CD34, CD11b, CD16b and CD14 expression. (**f**) Isotype controls (grey) for each antibody used. Freshly isolated human blood granulocytes and monocytes were used as positive controls (black). Expressions of indicated marker of neutrophilic progenitor at two time-points were illustrated as examples (orange/blue).

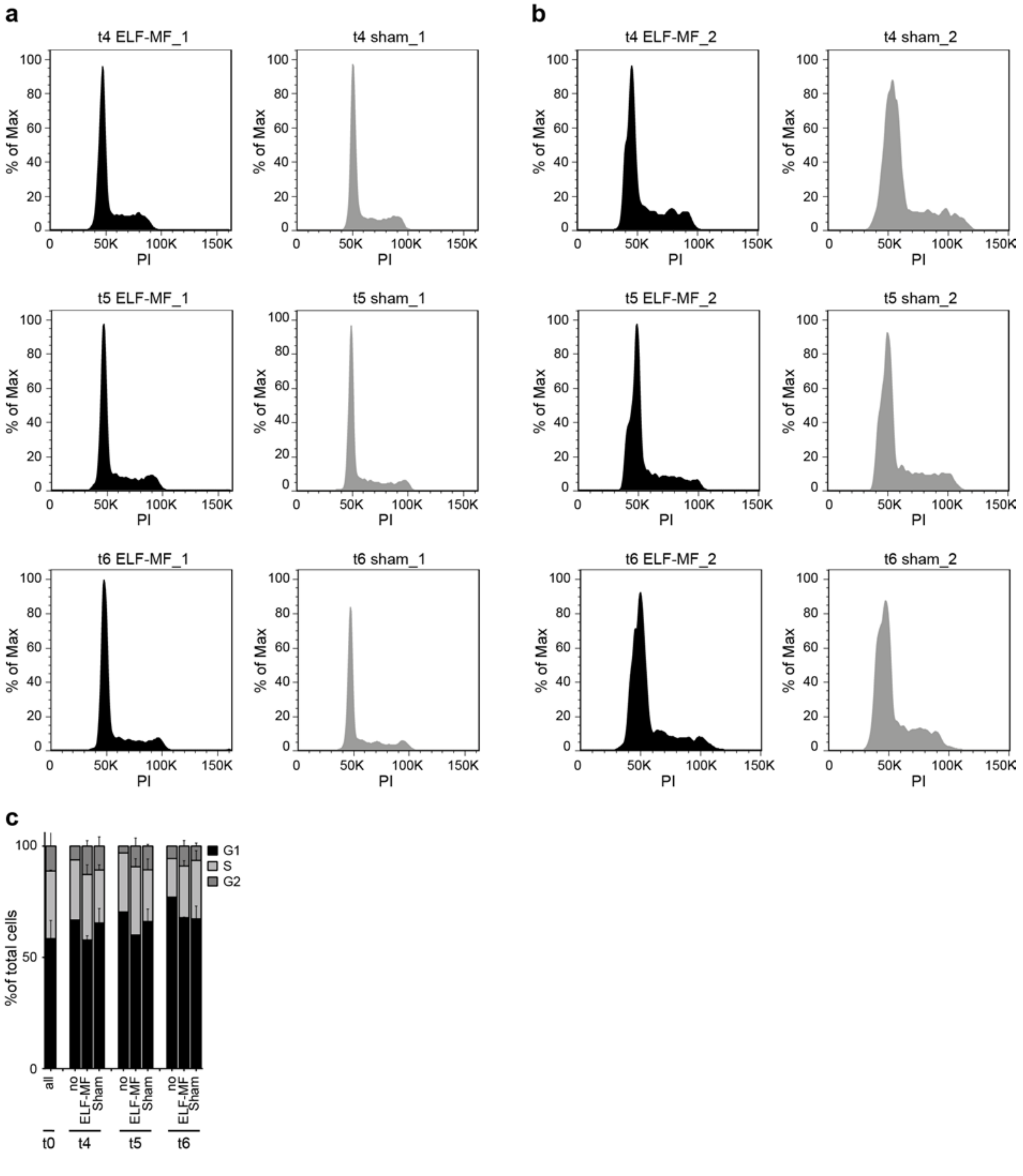

Supplementary Figure S7. Analysis of the cell cycle profiles in the differentiating neutrophilic cell population. Cell cycle profiles of two independent biological replicates (**a, b**) from neutrophilic progenitors, ELF-MF- (50 Hz powerline signal, 1 mT, 5' on/10' off) or sham-exposed, were analysed by flow cytometry 4, 5 and 6 days (t4, t5, t6) after induction of differentiation. (**c**) Summary and statistical analysis of cell cycle profiles. Mean percentage of cells in different cell cycle phases with SEM are shown. Data represent two biological replicates for ELF-MF and sham exposure, and one replica for the control differentiation. They were statistically analysed by the χ^2 test for each replicate (* $P < 0.05$).

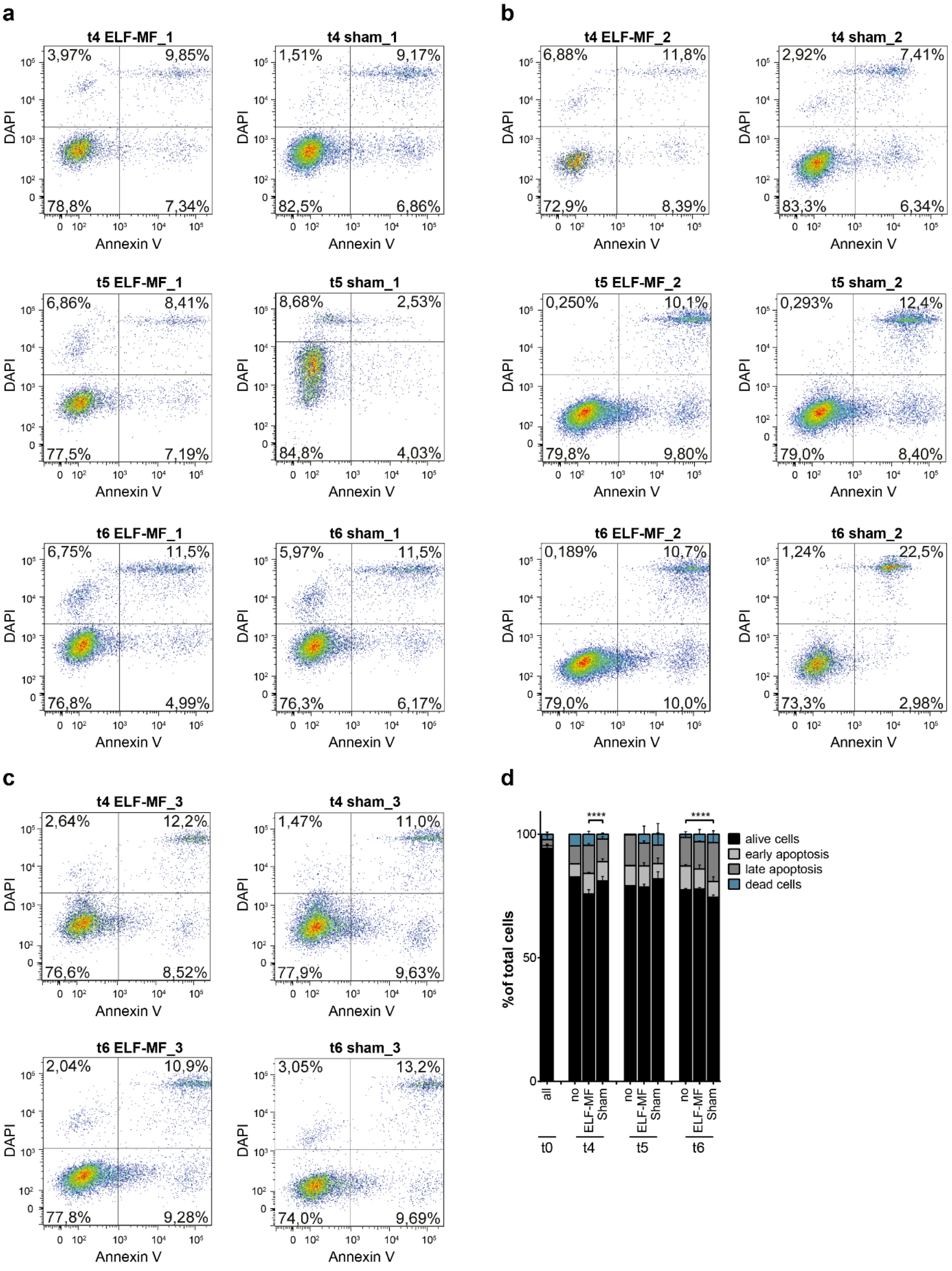

Supplementary Figure S8. Analysis of apoptosis in differentiating neutrophilic progenitors. Apoptosis in the haematopoietic cell population was analysed by flow cytometry before and at days 4–6 of the differentiation into neutrophilic lineage for non-exposed, ELF-MF- (50 Hz powerline signal, 1 mT, 5' on/10' off) and sham-exposed cultures. (**a–c**) Density plots with Annexin-V expression on the x-axis and DAPI permeability on the y-axis indicating alive cells (Annexin-V/DAPI double negative), early apoptotic (Annexin-V+/DAPI-), late apoptotic (double positive) and dead/necrotic (Annexin-V-/DAPI+) cells are shown for all biological replicates. (**d**) Summary and statistical analysis of the apoptosis, showing the mean percentage and SEM of cells in the different cell viability state at the indicated time-points. Data of three biological replicates (two for negative control and t5) were statistically analysed by pairwise comparisons with the χ^2 test (* $P < 0.05$), considering significance only when observed in every replicate.

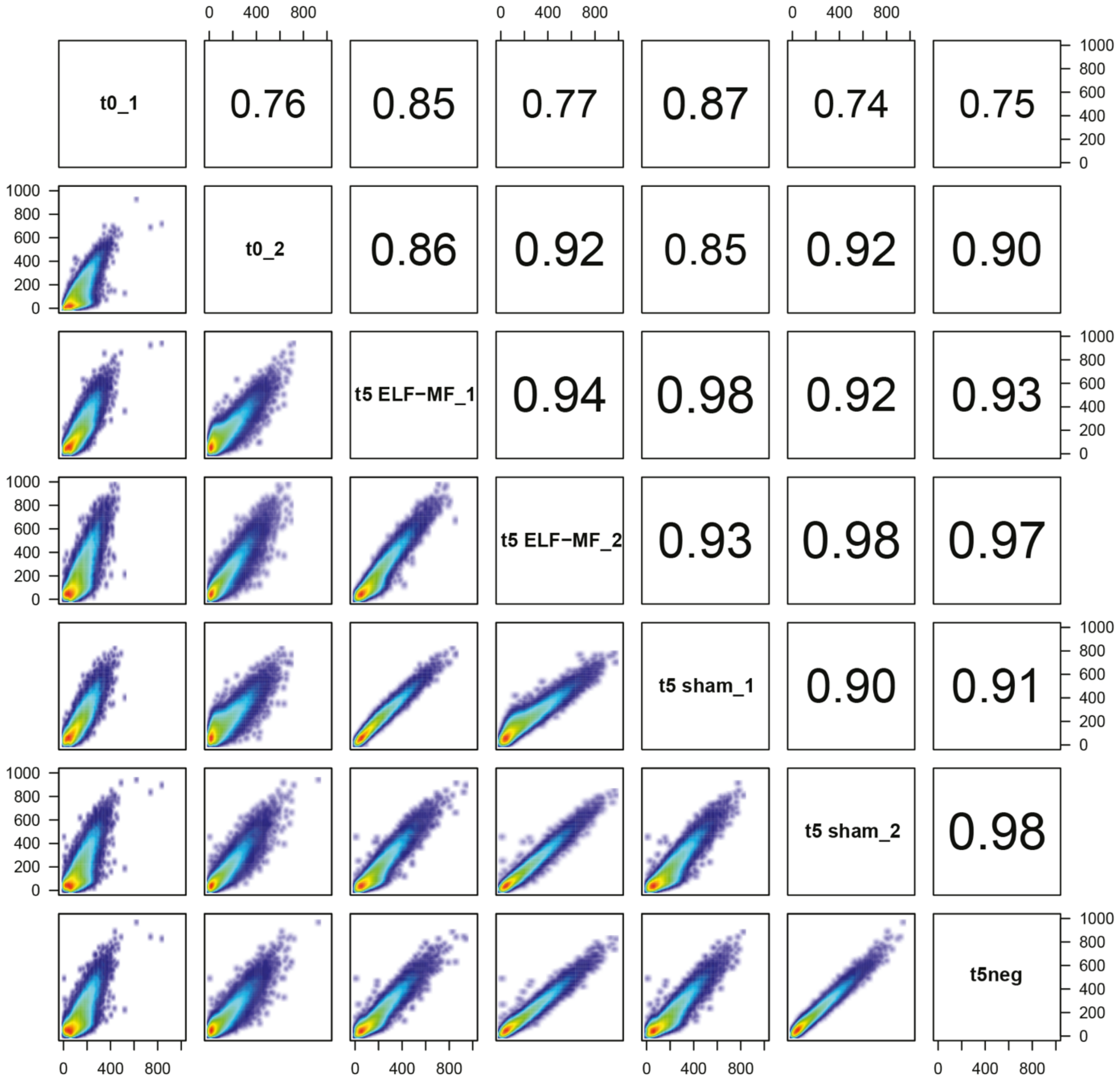

Supplementary Figure S9. Correlation between H3K4me2 ChIP-seq data of neutrophilic granulopoiesis. Global profiles of H3K4me2 histone modification of CD34+ cord blood cells (t0), ELF-MF- (50 Hz powerline signal, 1 mT, 5’ on/10’ off), sham- or non-exposed neutrophilic progenitor cells (t5) after 5 days of differentiation were generated by ChIP-sequencing. Two ChIP-seq replicates were generated for each condition, except only one replicate for the non-exposed neutrophilic progenitors (a pool of two independent biological replicates). The correlation of ChIP-seq reads in 500 bp genomic tiles between all H3K4me2 ChIP-seq samples is illustrated by density plots (lower left) and correlation coefficients (R values; upper right).

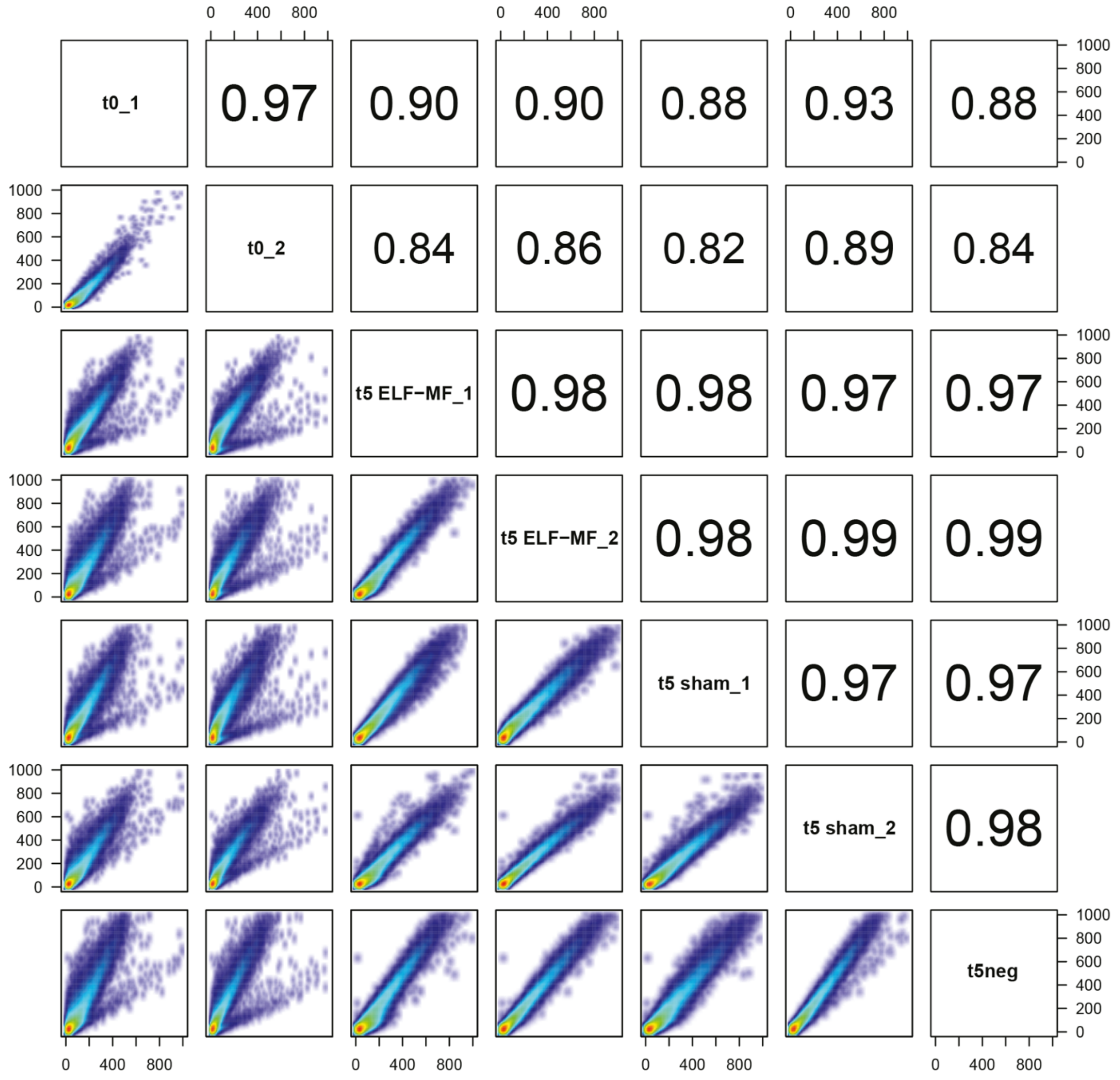

Supplementary Figure S10. Correlation between H3K27me3 ChIP-seq data of neutrophilic granulopoiesis. Global profiles of H3K27me3 histone modification of CD34+ cord blood cells (t0), ELF-MF- (50 Hz powerline signal, 1 mT, 5' on/10' off), sham- or non-exposed neutrophilic progenitor cells (t5) after 5 days of differentiation were generated by ChIP-sequencing. Two ChIP-seq replicates were generated for each condition, except only one replicate for the non-exposed neutrophilic progenitors (a pool of two independent biological replicates). The correlation of ChIP-seq reads in 500 bp genomic tiles between all H3K27me3 ChIP-seq samples is illustrated by density plots (lower left) and correlation coefficients (R values; upper right).

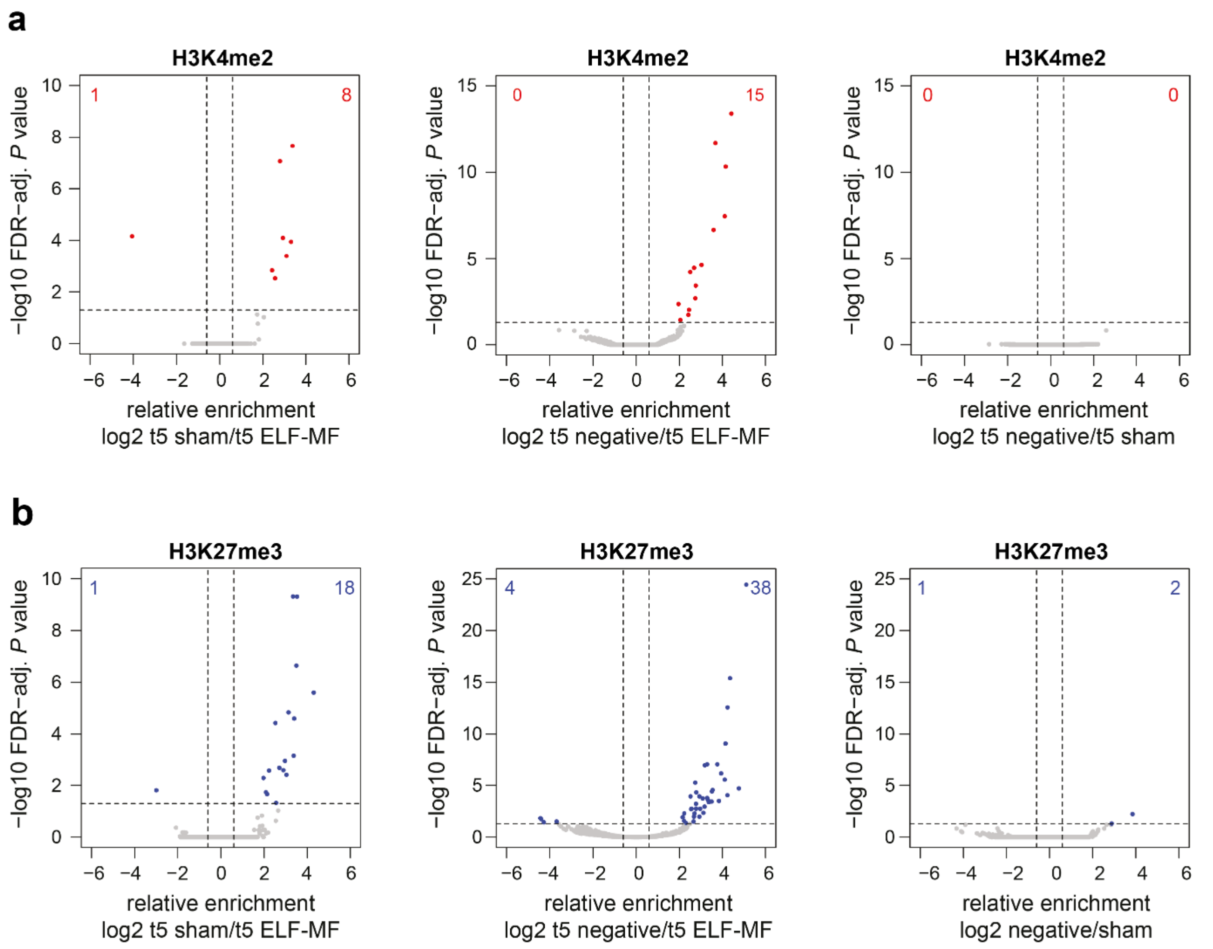

Supplementary Figure S11. Pairwise comparison of histone modifications in neutrophilic progenitors results in a few statistically significant alterations depending on the ELF-MF exposure condition. Human CD34+ cord blood cells were differentiated *in vitro* into the neutrophilic progenitor cells under ELF-MF (50 Hz powerline signal, 1 mT, 5' on/10' off), sham or no exposure for five days. H3K4me2 and H3K27me3 enrichment profiles for CD34+ cells and neutrophilic progenitors were generated by ChIP-seq and two replicates (one independent and a pool of two independent biological replicates, only one replicate for non-exposed neutrophilic progenitors) were statistically analysed. Differences in relative enrichment of ChIP-seq reads for H3K4me2 (**a**) and H3K27me3 (**b**) modifications within 500 bp genomic tiles are shown; log2-fold change (FC) (x-axis) between ELF-MF-, sham- or non-exposed neutrophilic progenitors are plotted against false discovery rate (FDR)-adjusted *P* value (calculated by likelihood ratio test with fixed dispersion) (y-axis). Statistically significant (FC > ±0.6, FDR-adjusted *P* < 0.05) tiles (500 bp or additional in 1,000 bp tiles) differentially occupied by H3K4me2 and H3K27me3 are highlighted in red and blue, respectively.

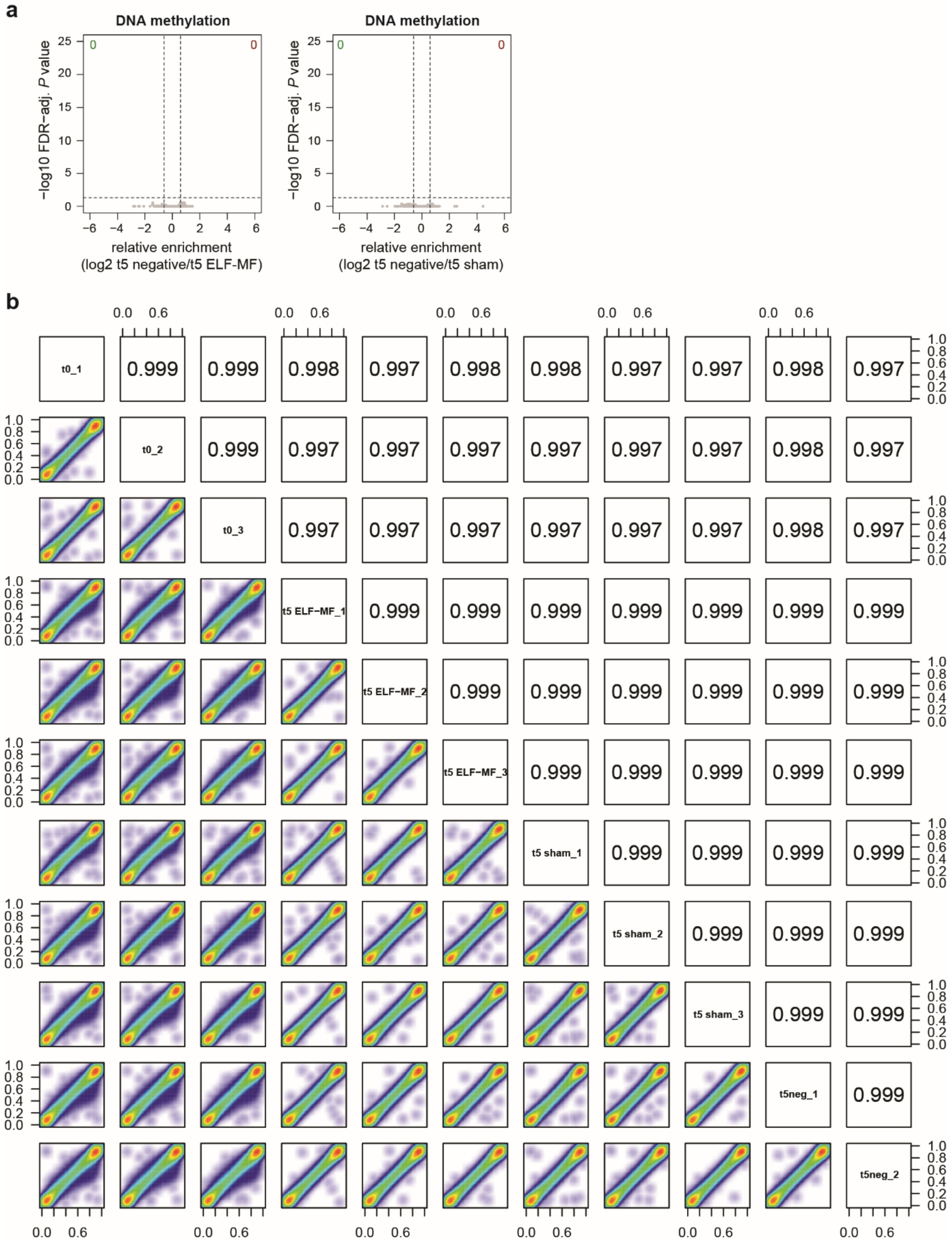

Supplementary Figure S12. Analysis of cytosine methylation data in neutrophilic differentiation. CD34+ cord blood cells were *in vitro* differentiated into the neutrophilic lineage for five days, while exposing them to ELF-MF (50 Hz powerline signal, 1 mT, 5' on/10' off) and sham or not exposed negative control conditions. DNA methylation of CD34+ cells (t0) and ELF-MF-, sham- or non-exposed neutrophil progenitor cells at day five (t5) was analysed by Illumina Infinium HumanMethylation 450 array. (**a**) Comparison of DNA methylation levels of negative controls with ELF-MF- and sham-exposed progenitor cells at day five, plotting relative differences in log2-fold change (FC) (x-axis) against false discovery rate (FDR)-adjusted *P* value (moderated *t*-statistics; n=3, n=2 for negative control) (y-axis). (**b**) The correlation of DNA methylation levels (β-values) between all replicates and experimental conditions is illustrated by density plots and correlation coefficients (R values).

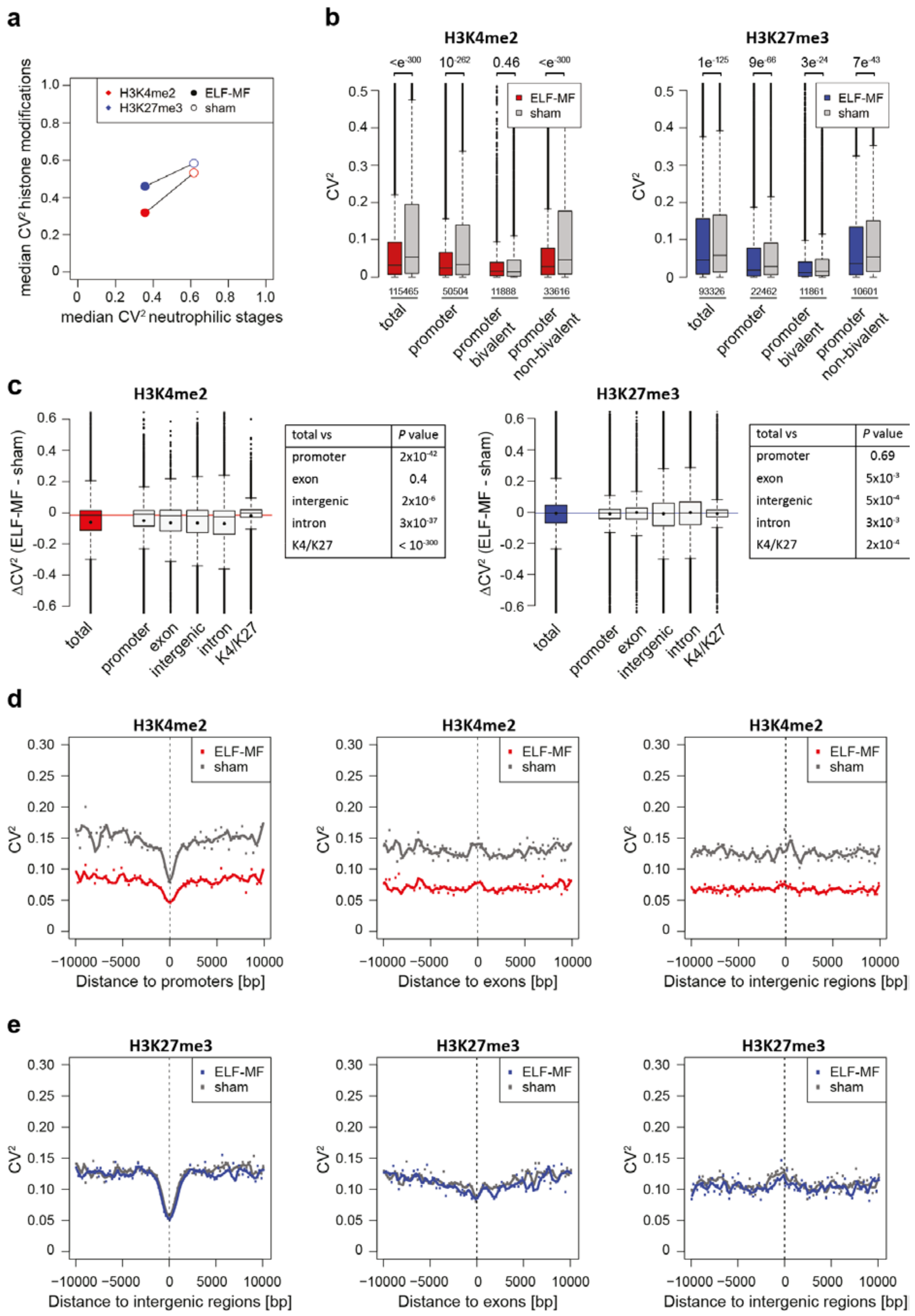

Supplementary Figure S13. Replicate variability of H3K4me2 and H3K27me3 marks in neutrophilic progenitors is locus-specific. Variability of ChIP-seq replicates for H3K4me2 and H3K27me3 modifications of ELF-MF- (50 Hz powerline, 1 mT, 5' on/10' off) and sham-exposed neutrophilic progenitors at day five of *in vitro* differentiation was assessed. (**a**) Median of the squared coefficient of variation (CV^2) of reads in 500 bp tiles of two H3K4me2 and H3K27me3 ChIP-seq replicates (x-axis) was correlated with the median variance of

neutrophilic stages (three replicates, y-axis) in ELF-MF- and sham-exposed neutrophilic progenitors. Neutrophilic stages were analysed by flow cytometry discriminating CD34+ cells (CD34+, CD11b-, CD16b-), promyelocytes (CD34-, CD11b-, CD16b-), myelocytes (CD34-, CD11b+, CD16b-) and metamyelocytes/neutrophils (CD34-, CD11b+, CD16b+). (**b–e**) The squared coefficient of variation (CV^2) of reads in 500 bp tiles of the two H3K4me2 and H3K27me3 ChIP-seq replicates was assessed with respect to genomic features: promoters (± 1,000 bp of TSS), exons, introns, intergenic regions (UCSC hg19) or bivalent domains. (**b**) Box-and-whisker plots illustrate median (lines) and mean (black circles) CV^2 values of H3K4me2 and H3K27me3 modification at non-bivalent (non-bi) and bivalent (bi) promoters. The *P* values above indicate significance levels by Wilcoxon rank sum test. (**c**) Box-and-whisker plots illustrate median (lines) and mean (black circles) delta CV^2 values (CV^2 ELF-MF – CV^2 sham) and horizontal lines indicate the median of all H3K4me2 or H3K27me3 tiles. The *P* values of the Wilcoxon rank sum test indicate significant differences between delta CV^2 values of all tiles and delta CV^2 values of tiles at corresponding genomic feature. (**d, e**) Comparing ELF-MF- and sham-exposed samples, mean variability of ChIP-seq reads of H3K4me2 (**d**) and H3K27me3 (**e**) tiles (CV^2 values on y-axis) were plotted as function of distance to the nearest corresponding genomic feature.

Supplementary Methods

Cell cycle analysis by propidium iodide staining

20–50 $\times 10^3$ cells were fixed with cold 70% ethanol overnight. Cells were collected by centrifugation, resuspended in 200 mM Tris-HCl pH 7.5, 200 mM NaCl and 1 mg/mL RNase A and incubated at 37°C for 30 min. After addition of 0.5 mg/mL pepsin (in 0.2% HCl) and incubation for another 15 min at 37°C, the cells were stained with propidium iodide (100 µg/mL in PBS pH 7.5) and incubated on ice for at least 30 min. The DNA content was measured with a FACS cytometer (BD Biosciences) in the FL2 channel (emission at 575 nm). Raw data were analysed with the FlowJo software (TreeStar). Data were statistically analysed by χ^2 test for each replica as well as by pairwise comparison by Student's *t*-test in GraphPad Prism.

Apoptosis measurement by Annexin-V staining

The number of apoptotic cells in the population was estimated by the Annexin-V Alexa488/PI kit (Invitrogen) according to the provider's recommendations. 20–50 $\times 10^3$ cells were blocked on ice with Annexin-binding solution supplemented with 1% bovine serum albumin for 15 min before staining with the FITC-anti-Annexin-V antibody at RT for 20 min, followed by three washing steps with Annexin-binding solution. PI (Invitrogen) or DAPI was added before analysing the cells. Samples were measured with a FACS cytometer (BD Biosciences) and analysed by the FlowJo software. Data were statistically analysed in GraphPad Prism by χ^2 test for each replicate as well as pairwise comparison by Student's *t*-test.

Identification of cell differentiation state by flow cytometry

Maturation status of the differentiating neutrophilic cell population was analysed by flow cytometry detecting cell surface markers (CD34, CD11b, CD16b) with antibodies. After blocking the cells in Annexin-binding solution (Invitrogen) with 1% BSA on ice for 20 min, the cells were stained in 50 µL blocking buffer on ice for 20 min with the following combination of antibodies: PE mouse anti-human CD16b, APC mouse anti-human CD34, APC-Cy7 mouse anti-human CD11b, PE-Cy7 mouse anti-human CD14 (all from BD Biosciences) and FITC-anti-Annexin-V (Invitrogen) and washed three times with

blocking buffer. As negative control, samples were stained with an unrelated isotype-matched antibody (BD Biosciences) (Supplementary Fig. S6f). Freshly isolated monocytes and granulocytes from human blood, separated by a Ficoll-plaque-plus density gradient (GE Healthcare), were used as positive controls. Cell samples were analysed by a FACSCanto II (BD Biosciences) (Supplementary Fig. S6d) and Flow cytometry data were determined with FlowJo software (TreeStar), with gating to exclude doublets and nonviable cells on the basis of pulse width and incorporation of DAPI in combination with Annexin-V. The neutrophilic differentiation stages were identified by gating on subpopulations according to the expression of surface markers (Supplementary Fig. S6e)[1]: CD34+ cells (CD34+, CD11b-, CD16b-), Promyelocytes (CD34-, CD11b-, CD16b-), Myelocytes (CD34-, CD11b+, CD16b-) and Metamyelocytes (CD34-, CD11b+, CD16b+). Monocytes were identified according to the expression of CD14+.

Chromatin-Immunoprecipitation (ChIP)

Proteins bound to DNA were crosslinked by incubating cells with freshly prepared 1% methanol-free formaldehyde in PBS pH 7.4 at room temperature under slow agitation for 10 min. Crosslinking was stopped by the addition of glycine to a final concentration of 125 mM. Cells were washed three times with ice-cold PBS, pelleted and snap-frozen. Cells were lysed in cold lysis buffer (1% SDS, 10 mM EDTA, 50 mM Tris-HCl pH 8 and 0.5% Triton-X100, 1 mM PMSF, 1× cOmplete™ Protease Inhibitor Cocktail [Roche]) on ice for 20 min while vortexing several times. To produce random chromatin fragments ranging from 250–400 base pairs in length, cell lysates were sonicated for 30 min (30 sec on, 30 sec off, power high) by a Bioruptor sonicator with a cooling system (diagenode) and cleared by centrifugation at 14,000×g at 4°C for 10 min. Chromatin concentration was estimated by measuring absorbance at 260 nm on a Nanodrop 1000 (Witec AG). For histone ChIPs, 20–30 µg chromatin was diluted 10-fold in ChIP dilution buffer (0.01% SDS, 16.7 mM Tris-HCl pH 8.0, 1.2 mM EDTA, 167 mM NaCl, 1.055% Triton X-100, 1 mM PMSF, 1× cOmplete™), saving 1% of the volume for input analysis. Diluted chromatin was pre-cleared with 20 µL of magnetic Protein G beads (Invitrogen) and pre-blocked with 1 mg/mL BSA and 1 mg/mL tRNA or single-stranded salmon sperm DNA at 4°C for 1 h, prior to incubation with 1–2 µg of the respective antibody (H3K4me2: 07-030 Millipore; H3K27me3: 07-449 Millipore) overnight at 4°C under slow rotation. Histone-antibody-complexes were pulled down by incubation with 40 µL of pre-blocked magnetic Protein G beads at 4°C for 2 h, followed by serial washing with 500 µL ChIP wash buffer I (150 mM NaCl, 20 mM Tris-HCl pH 8.0, 2 mM EDTA, 0.1% SDS, 1% Triton X-100, 1 mM PMSF), 500 µL ChIP wash buffer II (500 mM NaCl, 20 mM Tris-HCl pH 8.0, 2 mM EDTA, 0.1% SDS, 1% TritonX-100, 1 mM PMSF) and 500 µL ChIP wash buffer III (250 mM LiCl, 1% NP40, 10 mM Tris-HCl pH 8.0, 1 mM EDTA, 1% sodium deoxycholate, 1 mM PMSF) at 4°C under rotation. After two additional washes with 500 µL TE buffer (10 mM Tris-HCl pH 8, 1 mM EDTA), bound complexes were eluted by two sequential incubations with 250 µL elution buffer (1% SDS, 0.1 M $NaHCO_3$) at 65°C for 10 min while shaking at 1,400 rpm. Reversal of crosslinking in eluates and input samples was done by incubation at 65°C for 4 h in the presence of 200 mM NaCl. After proteinase K digestion (50 µg/mL) in the presence of 10 mM EDTA and 40 mM Tris-HCl pH 6.5 at 45°C for 1 h, DNA was purified by phenol/chloroform extraction and NaCl/ethanol precipitation and resuspended in 10 mM Tris-HCl pH 8.0.

Supplementary References

1 Elghetany, M. T. Surface antigen changes during normal neutrophilic development: a critical review. *Blood Cells Mol. Dis.* **28**, 260-274, doi:10.1006/bcmd.2002.0513 (2002).
